# Spontaneous Lesions of Endangered Geriatric Julia Creek Dunnarts (*Sminthopsis douglasi*, Archer 1979) with Emphasis in Reproductive Pathology

**DOI:** 10.3390/vetsci11040142

**Published:** 2024-03-22

**Authors:** Viviana Gonzalez-Astudillo, Andrea Schaffer-White, Lawrence Noble, Patricia O’Hara, Peter Murray, Tamsin S. Barnes, Rachel Allavena

**Affiliations:** 1 School of Veterinary Science, The University of Queensland, Gatton Campus, Gatton, QLD 4343, Australia; l.noble@uq.edu.au (L.N.); peter.murray2@usq.edu.au (P.M.); t.barnes@uq.edu.au (T.S.B.); r.allavena@uq.edu.au (R.A.); 2 Independent Veterinary Pathology, 3245 Logan Road, Underwood, Brisbane, QLD 4119, Australia; andrea.schafferwhite@ivpath.com.au; 3 School of Agriculture and Food Sustainability, The University of Queensland, Gatton Campus, Gatton, QLD 4343, Australia; patricia.ohara@uq.edu.au; 4 Biological Resources, The University of Queensland, Brisbane, QLD 4072, Australia; 5 School of Agriculture and Environmental Science, University of Southern Queensland, Toowoomba, QLD 4350, Australia

**Keywords:** aging, captive breeding, Dasyuridae, lymphoma, marsupial, senescence, reproductive pathology, *Sminthopsis douglasi*

## Abstract

**Simple Summary:**

This study focuses on the spontaneous lesions observed in a captive colony of geriatric Julia Creek dunnarts, an endangered carnivorous marsupial. Despite their conservation status, no prior research has focused on the conditions affecting captive individuals attaining senescence. We examined one wild and thirty-five captive-born, mostly elderly dunnarts that failed to reproduce over several breeding periods. From these, ten dunnarts had normal findings. Among females, the most common issue was cystic glandular hyperplasia (eight cases); cutaneous lesions were infrequent (two cases). Males showed testicular degeneration, aspermatogenesis, or atrophy (three cases). Cutaneous lesions compatible with epitheliotropic T cell lymphomas were observed in both sexes (five cases) and thus, an underlying oncogenic viral etiology is suspected. This is the first study that documents spontaneous diseases in aging Julia Creek dunnarts, shedding light on geriatric conditions within a conservation context.

**Abstract:**

Julia Creek dunnarts are an endangered species of carnivorous marsupials and the focus of multiple conservation strategies involving significant resources such as captive breeding programs. Despite the relevance for conservation, no study to date has focused on evaluating geriatric diseases in dunnarts. This study describes the pathology findings in a group of one wild and thirty-five captive-born, mostly geriatric Julia Creek dunnarts that failed to produce offspring over multiple breeding periods. A total of 20 females and 16 males were submitted for a postmortem examination, with ages ranging from 9 to 42 and 12 to 42 months for females and males, respectively. Of these, 10 had unremarkable findings. The most common condition in females was cystic glandular hyperplasia (n = 8), typical of hormonal dysregulation profiles in senescence, particularly hyperestrogenism. Rarely, cutaneous disease represented by unidentified dermal round cell infiltrates was observed in females (n = 2). Primary reproductive hormonal dysregulation was also suspected in males diagnosed with testicular degeneration, aspermatogenesis and/or atrophy (n = 3). Cutaneous round cell infiltrates, possibly compatible with epitheliotropic lymphomas, were seen in males (n = 3), and 2/3 affected males also had concurrent testicular degeneration or atrophy, indicating male sex could be a predictor for lymphoid neoplasia in aged dunnarts, especially in individuals with concurrent testosterone-luteinizing hormone dysregulation as it occurs in gonadectomized animals. The role of an underlying viral etiology is also explored. This study is the first to describe major spontaneous diseases in endangered aged Julia Creek dunnarts, providing an important understanding of senescence and geriatric diseases within a conservation context.

## 1. Introduction

Captive breeding programs have been used to counteract wildlife population declines, maintain gene pool diversity, achieve threat mitigation and reintroduce future self-sustaining populations. Such programs have been key in the recovery of multiple species such as the Arabian oryx (*Oryx leucoryx*), California condor (*Gymnogyps californianus*) and the golden lion tamarin (*Leontopithecus rosalia*), amongst others [1,2,3]. However, captive breeding is considered a last resort conservation tool due to the inexorable behavioral, phenotypical and genetic challenges faced by wildlife in captive environments. Successful establishment of captive breeding populations is challenging and requires careful planning to evoke an appropriate environment to achieve successful reproduction, major logistics, long-term financial and administrative continuity as well as avoiding domestication. These conditions are necessary to secure the maintenance of healthy individuals and ensure successful reintroductions and long-term maintenance of genetically viable populations.

Captive breeding has been one of the conservation tools used to secure healthy breeding stocks of Julia Creek dunnarts (*Sminthopsis douglasi*), a small nocturnal Australian carnivorous marsupial of the family Dasyuridae endemic to the state of Queensland. Longevity is similar to other small marsupials; wild male and female Julia Creek dunnarts live up to 12 and 24 months, respectively [4], with records extending to three years in captivity. Typically, they are found in low densities [5] and their habitat includes grassland ecosystems with heavy clay soils and a rich diversity of invertebrate and vertebrate prey [4]. The species was considered extinct in the 1980s and was re-discovered from owl pellets, cat remains and by the capture of live specimens a decade later [6,7]. Currently, Julia Creek dunnarts are listed as vulnerable under the Environment Protection and Biodiversity Conservation Act 1999 and Endangered under the Nature Conservation Act 1992 in Queensland [5], with feral cats (*Felis catus*) and invasive weeds (e.g., prickly acacia, *Vachellia nilotica*) outcompeting understory vegetation and heavy livestock grazing as important threats [8]. Additional threats include extreme weather from climate change, as Julia Creek dunnarts abundance patterns are positively correlated to rainfall, which directly influences understory cover growth, which in turn provides shelter from potential predators [4]. Significant resources have been invested to bring forward a conservation plan for the species [9] which does not include disease management despite the documented predisposition of dasyurids to proliferative diseases [10]. Thus, diseases are not currently considered a threat to the species and no active measures to investigate or prevent the occurrence of common diseases have been proposed. 

Similar to other wildlife managed in captive environments, Julia Creek dunnarts are vulnerable to potential disease outbreaks and age-related diseases as they easily outlive wild conspecifics. Due to the direct impact of spontaneous geriatric diseases on their conservation, this manuscript focuses on documenting the reproductive and non-reproductive lesions observed in geriatric Julia Creek dunnarts held in a captive breeding colony.

## 2. Materials and Methods

### 2.1. Population and Data Collection

The colony was initially formed in the 1990s following the capture of eight wild-caught founder individuals that were held at La Trobe University prior to their transfer to the Pearcedale Conservation Park (PCP) in Victoria [6,9], where an additional eight wild-caught dunnarts from Bladensburg National Park were transferred to the captive colony in April 2001 [6]. In August 2002, 12 dunnarts (3 M:9 F) were transferred from PCP to David Fleay Wildlife Park (DFWP) on the Gold Coast in Queensland, Australia. In August 2006, a male was introduced into the captive colony and was the last wild-caught dunnart to be introduced into the colony. The captive colony was transferred from DFWP to The University of Queensland (UQ) Native Wildlife Teaching and Research (NWT&R) Facility on the Gatton Campus in March 2009 (Animal Ethics permits SAS/930/08/BREED, SAFS/441/11/BREED; Queensland Government permit WIEP05809909). 

Reproductive management of the captive colony at UQ involved monitoring female dunnarts for signs of estrus (increased presence of epithelial cells in urine, running wheel activity and feed residue) and were paired for breeding. Following the placement of the male in the female’s enclosure (n = 298), mating occurred in approximately one-third of the introductions from 2009 to 2014. A total of 11 litters (47 joeys) were produced at the NWT&R Facility with 98% surviving to weaning.

The colony in the UQ NWT&R Facility rapidly attained senescence with multiple individuals reaching up to 3.5 years (lifespan 1–2 years) failing to produce offspring, despite observed matings across multiple breeding seasons. The absence of new genetics into the colony contributed to inbreeding depression, resulting in the disbanding of the colony in early 2014. Dunnarts eligible for inclusion in this study were found dead aged >1 year or euthanized due to age and/or signs of disease (e.g., progressive loss of body condition, abdominal distension, lethargy, alopecia), which failed to reproduce upon reaching senescence and received postmortem examination. There were two subsets of dunnarts. The first subset included those that had received a postmortem examination and follow-up histopathology at the time of death/euthanasia (1991–2014, n = 17). Detailed postmortem examinations were performed in 10 cases and histopathology was conducted in sections of formalin-fixed tissues received in an additional seven cases from offsite autopsies. Relevant case material was procured from archived samples from the UQ Pathology Department. The second subset comprised those that had died/been euthanized between 2009 and 2012, preserved at −20 °C (n = 19), and autopsied after a slow thawing process at 4 °C in April 2023. Details about origin, postmortem workup, broad lesion category and demographics are included in Supplementary Table S1. 

### 2.2. Gross Examination, Sample Collection and Processing

A detailed postmortem examination was conducted in each dunnart on the first subset following UQ’s standard operating procedures. Representative samples of main organs such as brain, lungs, heart, liver, spleen and/or lymph nodes, stomach, intestine, kidneys, urinary bladder, skeletal muscle, reproductive tract (ovaries, uteri, cervix and vagina, testes and prostate) were collected and fixed in 10% buffered formalin prior to routine histopathology processing, to be paraffin-embedded, sectioned at 4 um, mounted on glass slides, and stained with hematoxylin and eosin (H&E) stain. Due to the expected severe freezing artefact and lack of funding to cover for histopathology of all main organs, histopathology was only carried out in the reproductive tract and skin in the second subset of dunnarts showing gross lesions. 

### 2.3. Ancillary Testing

Immunohistochemistry (IHC) methods for markers targeting pan-leukocytic cellular populations (CD68), T-lymphocytes (CD3), B-lymphocytes (CD20, CD79a, Pax-5), histiocytic cells (Iba-1), hematopoietic tissue (CD45 LCA) or epithelial cells (AE1/AE3) were carried out according to published protocols [11]. Details about IHC protocols can be found in Supplementary Material S1. Photomicrographs obtained during the validation procedures for each marker can be found in Supplementary Figures S1–S8. Histochemistry (Gram, Toluidine blue) was performed in a case-by-case basis following UQ’s and external laboratory standard laboratory protocols.

## 3. Results

Main demographic data, reproductive tract and significant non-reproductive tract morphological diagnoses are included in Table 1 and Table 2 and in Supplementary Table S1. A total of 36 dunnarts from the colony were submitted for a postmortem examination: 20 females and 16 males from 2009 through 2022. From these, five geriatric males, one adult non-geriatric male and four adult, non-geriatric females were excluded from analyses as their gross and histopathological examinations were unremarkable or had non-reproductive lesions (e.g., cryptococcosis in male dunnart 127) or their histopathology examination was outsourced (fibrosarcoma in the hindlimb of male dunnart 176). Few dunnarts had good to excellent (n = 5) or adequate/moderate body condition (n = 6). Poor body condition was recorded in nine dunnarts. Body condition was not disclosed in the clinical history of five dunnarts. Weight was recorded in the second subset of autopsies with dunnarts being in normal to excellent body condition when weighing around >52 g (n = 9), whilst those in poor body condition weighing often <50 g (n = 10).

For the second subset of dunnarts in the present study (n = 14), there was a similar mating success (33.33% from 168 introductions) in the colony. Only two litters (six joeys) were produced, resulting in a lower birthing success; however, all six offspring survived to weaning.

### 3.1. Gross Pathology—Female Reproductive Tract

**Figure 1:**
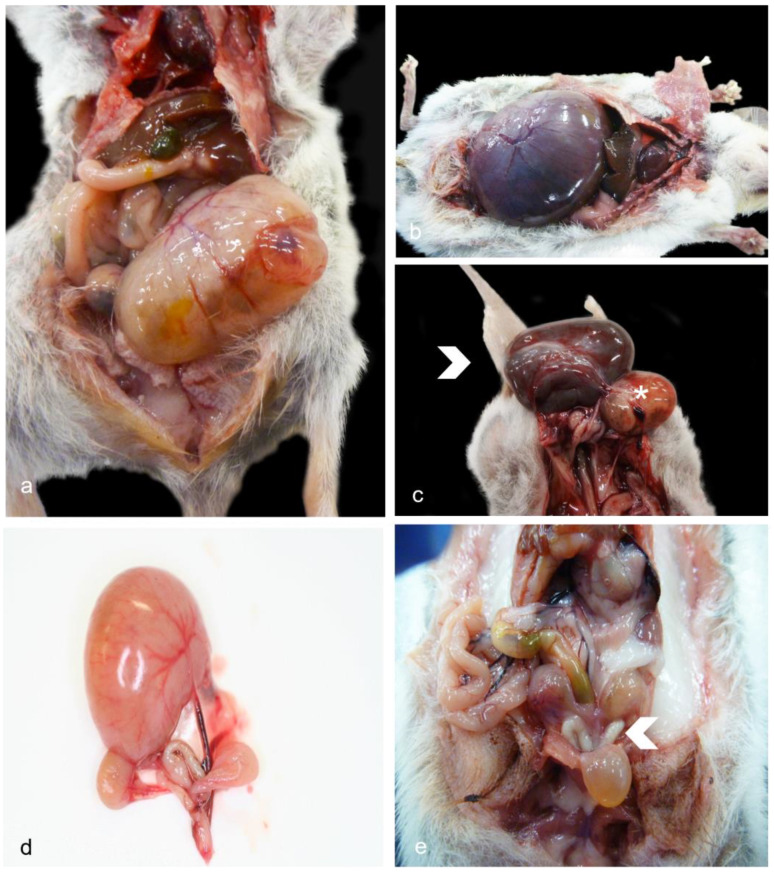
**Gross female reproductive lesions in geriatric Julia Creek dunnarts.** (**a**) **Cystic glandular hyperplasia (CGH), dunnart 14.** A 2.5 cm diameter, pale pink to tan, firm abdominal cyst filled by a pale tan fluid expands the cavity and displaces abdominal viscera. (**b**) **CGH, dunnart 11.** The right uterus measures 3.5 cm in diameter, is diffusely dark purple, firm and is found expanding the abdominal cavity, displacing and compressing the abdominal viscera cranially as well as compressing the thoracic cavity, causing pulmonary collapse. The uterine mass is filled with approx. 5 mL of dark red, inspissated fluid. (**c**) **CGH and endometrial papillary adenoma, dunnart 13.** Rarely, a single uterus is expanded by two separate masses; the largest mass measures 2.5 cm diameter (arrowhead) and corresponds to CEH. The second mass (asterisk) is an endometrial papillary adenoma, measuring about 2 cm in diameter and filled by viscous dark brown-orange material. (**d**) **Ovarian cyst, Not Otherwise Specified—NOS, dunnart 6.** The ovary appears to be expanded by a single, unilocular mass filled by light yellow-pink fluid; bilaterally, the uteri are appreciated below the mass. Photo credit: Peter Moore. (**e**) **Vaginal neutrophilic infiltrates, dunnart 10.** Grossly, the lateral vaginas (arrowhead) are discolored off-white and moderately expanded, possibly indicating underlying pathologic inflammation. However, physiologic neutrophilic infiltrates that occur in certain small mammal species during metestrus and diestrus cannot fully be ruled out. This dunnart also had CGH and a cystic oviduct (not on image).

Lesions were often found affecting the uteri, mainly represented by cystic glandular hyperplasia (CGH, Figure 1a–c), and less frequently by cysts affecting other parts such as the oviduct or ovaries (Figure 1d). Reproductive inflammation was uncommon and was mostly represented by neutrophils (Figure 1e). No cases of malignant neoplasms were observed. Grossly, when the reproductive tract was affected by cystic disease, lesions were more often evident in the endometrium (dunnarts 3, 7–8, 10–11, 14, 24, 27), rarely accompanied by cystic expansion of adjacent anatomy such as the oviduct (cystic oviduct; dunnart 10) or ovary only (ovarian cyst Not Otherwise Specified—NOS; dunnart 6). Ovarian cyst NOS were described as having unilateral severe distension, and the oviducts as having one enlarged 5–7 mm diameter, light yellow-pink, soft, fluid-filled cyst each mostly in the proximal end. No macroscopic lesions were observed in the external genitalia in any of the females in this study. In general, and for those individuals for which information was recorded, disease of the reproductive tract in females was more often unilateral (n = 14) in comparison to bilateral (n = 3).

### 3.2. Histopathology—Female Reproductive Tract

**Figure 2:**
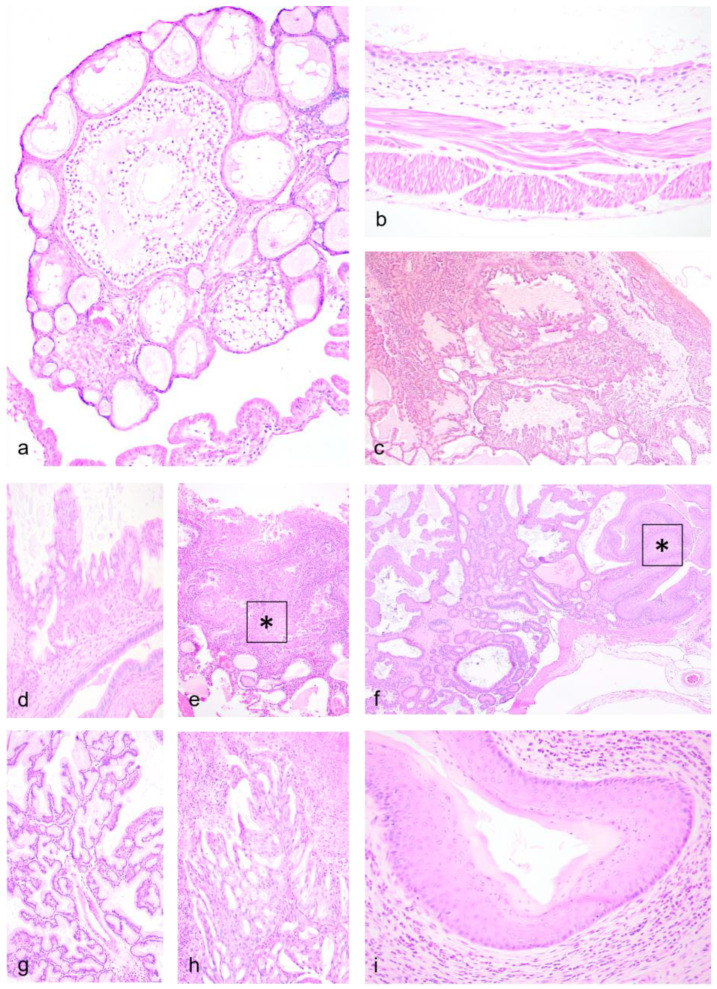
**Female reproductive lesions in geriatric Julia Creek dunnarts.** (**a**) **Age-related ovarian atrophy, dunnart 8.** Low numbers of primordial or primary follicles, few secondary follicles and 2 corpora albicans are observed. No corpora lutea are present, potentially indicating acyclic state. Hematoxylin and eosin (H&E) stain, 10×. (**b**) **Cystic oviduct, dunnart 9.** The proximal end of the oviduct (ampulla or isthmus region) is expanded by a single, 5–7 mm diameter; fluid-filled cyst lined by a single layer of ciliated attenuated columnar epithelium; H&E stain, 40×. (**c**) **Cystic glandular hyperplasia (CGH), dunnart 3.** Subgross view of the uterine wall with diffusely, actively proliferating, tortuous, dilated and often cystic endometrial glands. Hyperplastic glands lie back-to-back, supported by stromal bands of variable width and segmentally contain papillae covered by columnar, stratified to pseudostratified epithelium. H&E stain, 4×. (**d**) **CGH with mucin, dunnart 7.** High magnification view into one of the cystic structures found expanding the endometrium, formed by anastomosing trabeculae of thin fibrous connective tissue septa lined by a single line of ciliated columnar secretory epithelium, with abundant goblet cells; H&E stain, 20×. (**e**) **CGH with squamous metaplasia, dunnart 11.** The endometrium is expanded by numerous islands and anastomosing trabeculae forming cysts filled by fluid and necrotic debris lined by tall columnar cells. Foci of metaplastic, stratified, squamous, non-keratinizing epithelium are observed (asterisk). H&E stain, 20×. (**f**) **Endometrial (glandular) polyp, dunnart 14.** A polypoid mass protrudes into the uterine lumen comprising numerous glands lined by predominantly hyperplastic/dysplastic columnar glandular epithelium, mimicking endometrial mucosa, and supported by a stroma comprising spindle cells with variable amounts of collagen and scattered small-caliber vasculature. Focal squamous metaplasia (asterisk) is also observed. H&E stain, 4×. (**g**,**h**) **Endometrial adenoma, papillary, with focal squamous metaplasia, dunnart 13.** A neoplastic mass supported by a broad base formed by well-differentiated columnar, pseudostratified, mucus-secreting epithelial cells arranged in a papillary pattern (**f**) and mimicking endometrial epithelium is observed replacing the uterine stroma. No invasion to the endometrium or myometrium is observed. Focal squamous stratified and pseudostratified non-keratinizing epithelium is also observed forming fronds, covered by large amounts of necrotic debris. H&E stain, 20×. (**i**) **Vaginal neutrophilic infiltrates, dunnart 10.** Florid neutrophilic infiltrates are observed multifocally performing exocytosis into the vagina mucosa and infiltrating the lamina propria, possibly supporting a pathologic vaginitis over those granulocytic infiltrates of physiological origin that infiltrate during metestrus or diestrus. H&E stain, 10×.

CGH was the most commonly diagnosed finding (Figure 2c–f) and was characterized by numerous proliferating, often cystic, endometrial glands. The stroma was reduced in comparison with the lesser affected portions of the endometrium. Additionally, in two dunnarts with CGH, anovulatory ovaries were found, deemed to be age-related atrophy cases (Figure 2a). CGH with concurrent squamous metaplasia was observed in four cases (Figure 2e), usually observed as focal lesions only co-occurring within CGH tissue. Endometrial (glandular) polyps were found in three cases (Figure 2f), containing numerous endometrial-like glandular profiles. Although oviductal lesions were uncommon in this study, a cystic oviduct was observed (Figure 2b) causing luminal expansion and degeneration of the oviductal mucosa. A single case of an endometrial adenoma was observed (Figure 2g,h) and was diagnosed as a papillary type, with numerous mucin-producing cells resulting in the production of large amounts of mucin. Findings in the cervix and vagina were infrequent (two cases) and were represented by neutrophilic infiltrates, one case along fibrosis (Figure 2i).

### 3.3. Additional Significant Non-Reproductive Disease—Female Dunnarts

From the second subset, two dunnarts presented cutaneous round cell infiltrates: dunnarts 30 and 33. In detail, dunnart 30 had a cystic glandular uterine polyp and also presented with bilaterally, markedly thickened skin with alopecia and with focal, asymmetrical/unilateral erosions grossly, which histologically corresponded to a moderate proliferation of round cells lacking epitheliotropism but infiltrating and separating the dermal collagen in an equivocal sheet pattern. Dunnart 33 did not have concurrent reproductive lesions and was diagnosed instead with deep to mid-dermal round cell infiltrates that were not immunolabeled by any of the immunohistochemical markers used, as well as with cutaneous erosions and severe alopecia.

### 3.4. Gross Pathology—Male Reproductive Tract

**Figure 3:**
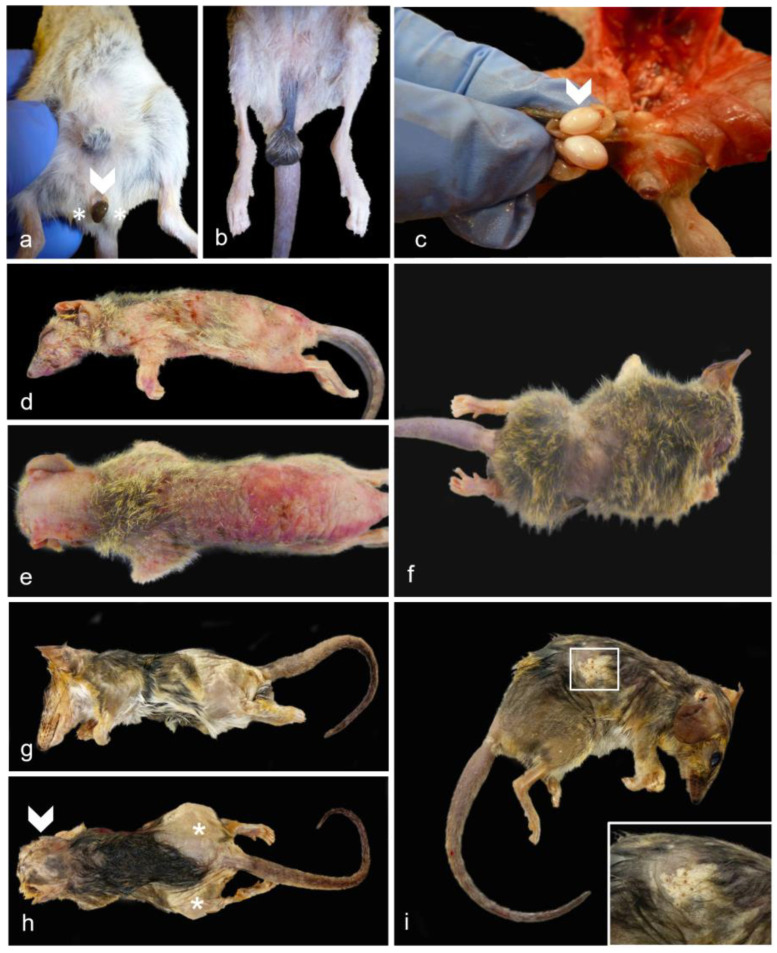
**Gross male reproductive and non-reproductive lesions in geriatric Julia Creek dunnarts.** (**a**) **Testicular degeneration/atrophy, dunnart 9.** The scrotal sac has a markedly shortened inguinal canal, turning it into a sessile structure with no obvious palpable testes, easily exposing the urogenital sinus (arrowhead) and bulbourethral glands (asterisks). (**b**) **Normal testes and scrotum, control dunnart.** Scrotum contains palpable testes and is suspended from the abdomen by a regular stalk with the inguinal canal containing the spermatic cord, completely covering the urogenital sinus and partially the bulbourethral glands. (**c**) **Bilateral testicular degeneration/atrophy, dunnart 4.** The left testicle (arrowhead) is around 1.5 times smaller than the globally larger right testicle, indicating a unilateral testicular tubular degeneration/atrophy in this dunnart. (**d**,**e**) **Cutaneous epitheliotropic lymphoma, dunnart 4.** Extensive areas of alopecia affecting 90% of the body with diffusely markedly thickened skin that is mottled pink, red and with multiple scabs and flaking in the caudo-dorsal region. The coat is maintained in the distal cervical and proximal dorsal and flank regions. (**f**) **Dermatitis with alopecia, dunnart not included in study included for comparison.** An approx. 2 cm band of circumferential alopecia is observed in the cranial abdomen. Follicular atrophy was noticed on histopathology. Note that the skin is smooth and not crusty, ulcerated or thickened, as in the epitheliotropic lymphoma cases. (**g**) **Telogen alopecia, dunnart 25.** Lateral view. Regionally extensive areas of alopecia are observed predominantly affecting the lateral thighs and rump region in this view. The skin appears thickened and focally wrinkly. (**h**) **Telogen alopecia, dunnart 25.** Dorsal view. About 50% of the body presents a bilaterally symmetrical alopecia, particularly focused in the head (arrowhead), lateral thighs and rump region (asterisks). (**i**) **Telogen alopecia, dunnart 31.** There is bilaterally symmetrical alopecia affecting the lateral thighs, rump and craniodorsal abdominal region. **Inset:** Alopecia was occasionally observed along comedone formation. The right pinna has been removed postmortem from all dunnarts in the photographs for DNA analysis.

Grossly visible lesions were only seen in the testes and scrotum. No gross lesions were observed for the epididymis, ductus deferens, bulbourethral glands or penis. Main lesions observed were primarily degenerative, represented by testicular degeneration/atrophy (Figure 3a), and causing a dramatic reduction in the length of the inguinal canal compared to a control (Figure 3b). Asymmetrical testes were noted in two cases, one of which was confirmed as bilateral testicular tubular degeneration/atrophy, likely in combination with autolysis (Figure 3c). For those individuals in which information was recorded, reproductive disease was more often unilateral (n = 3) in comparison to bilateral (n = 1) as seen in females.

### 3.5. Histopathology—Male Reproductive Tract

**Figure 4:**
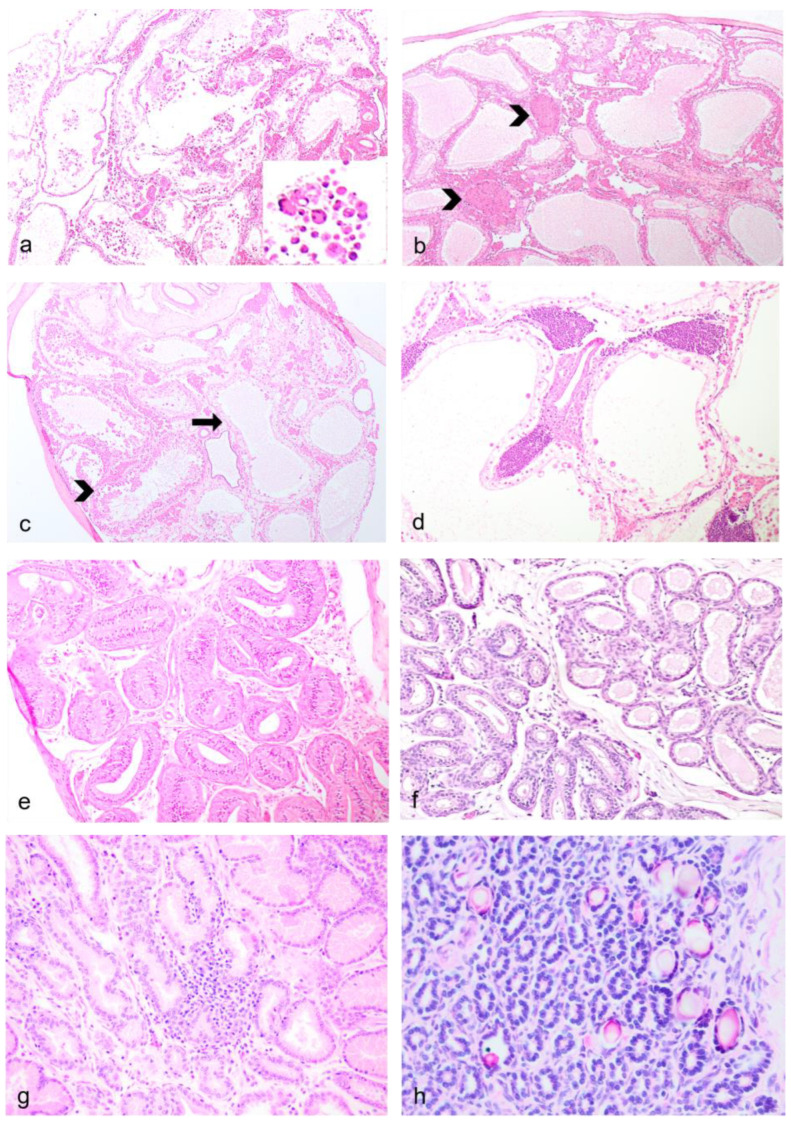
**Male reproductive lesions in geriatric Julia Creek dunnarts.** (**a**) **Testicular diffuse testicular tubular degeneration/atrophy with azoospermia, left testis, dunnart 4.** Despite autolytic artefact, which has removed the germinal epithelium and Sertolli cells from the seminiferous tubular lining, there are multiple, rounded, variably sized cells that are observed floating within the tubular lumina. Hematoxylin and eosin (H&E) stain, 4×. **Inset:** Small aggregates of large, rounded multiple-germ-cell nuclei of similar maturity are observed within degenerated seminiferous tubular lumina, interpreted as multinucleated giant cells of germ cell origin. (**b**) **Testicular degeneration with azoospermia, right testis, dunnart 4.** The seminiferous tubules are devoid of early and late spermatids; no spermatogenesis is observed. Supporting basement membrane is wavy, buckled and thickened. Few granulomas are observed replacing seminiferous tubules (arrowheads), likely resulting from tubular shrinkage and collapse and/or spermiostasis. Numerous peritubular interstitial (Leydig) cell aggregates are observed forming non-compressive clusters, likely enhanced by the disappearing tubules. Increased numbers of interstitial cells are a normal finding in multiple marsupial species. H&E stain, 4×. (**c**) **Segmental tubular degeneration/atrophy, dunnart 4.** Degeneration/atrophy also affected segments of seminiferous tubules, resulting in the visualization of tubules still containing germ cells in various stages of maturation (arrowhead) abutting others virtually devoid of germ cell epithelium but with a few remaining Sertolli cells (arrow). H&E stain, 4×. (**d**) **Metastatic testicular lymphoma, dunnart 5.** The interstitium is unilaterally expanded by dense aggregates of lymphocytes, mainly within vasculature. H&E stain, 10×. (**e**) **Epididymal segmental ductal atrophy, dunnart 5.** Segmental narrowing of the ductal lumina is observed with normal appearing epithelium, along occasionally lower epithelial height, likely due to testicular degeneration/atrophy in ipsilateral testis. H&E stain, 10×. (**f**) **Severe bilateral testicular atrophy, dunnart 8.** No identifiable testicular tissue was found within the scrotum, only a portion of the epididymis. Some of these tubules contain protein; however, epithelium is attenuated and no sperm is visualized. H&E stain, 20×. (**g**) **Prostatitis, dunnart 12.** The interstitium is multifocally expanded by small aggregates of lymphocytes and occasional plasma cells. H&E stain, 20×. (**h**) **Calcified prostatic concretions, dunnart 9.** Multifocally within glandular lumina, there are variably sized, irregularly shaped mineral deposits. H&E stain, 40×.

Testicular tubular degeneration/atrophy with aspermatogenesis (Figure 4a–c) were diagnosed in two males, one of which also had a primary cutaneous epitheliotropic lymphoma resulting in a splenic, pulmonary and unilateral testicular metastasis (Figure 4d). In these cases, common features included tubules being either segmentally/partially or diffusely depleted from germ cells, and lined by the highly resistant Sertoli cells, where postmortem preservation was adequate. Exfoliated individualized or multinucleated germ cells were observed within tubules containing some viable germ epithelium. In cases of either bilateral testicular atrophy (Figure 4e) or absence of palpable intra-scrotal testes (Figure 4f), degenerative lesions were also observed in the epididymis. Lesions in the prostate were mild and represented by interstitial inflammatory infiltrates (Figure 4g) or intratubular calcified concretions (Figure 4h).

### 3.6. Additional Significant Non-Reproductive Disease—Male Dunnarts

**Figure 5:**
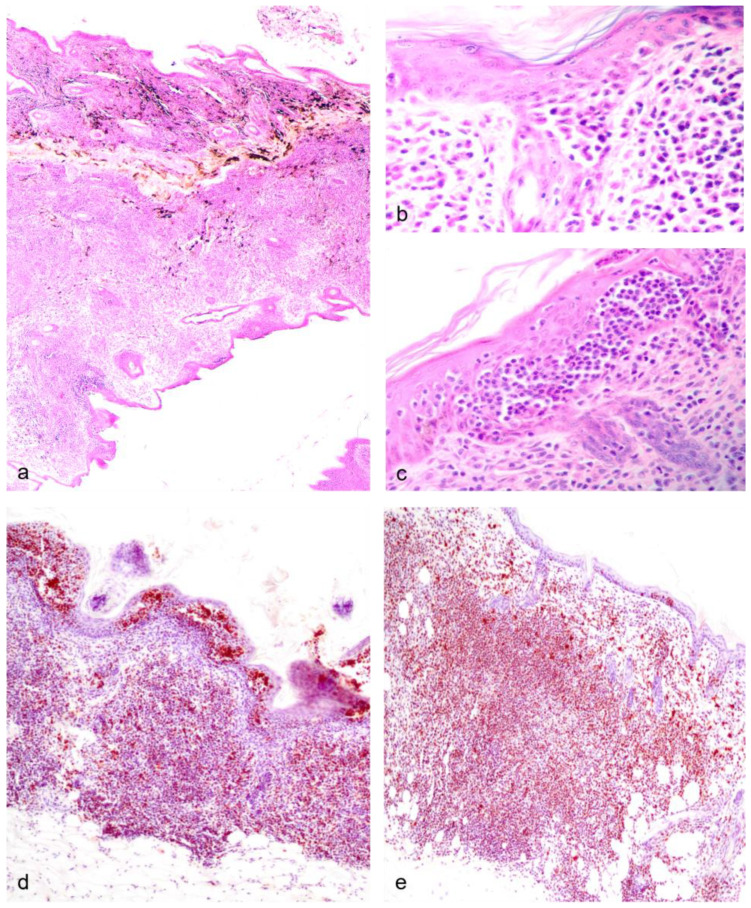
**Non-reproductive lesions of geriatric male Julia Creek dunnarts.** (**a**) **Primary cutaneous epitheliotropic lymphoma (mycosis fungoides), dunnart 5.** The dermis is infiltrated and replaced by a non-encapsulated and densely cellular neoplasm arranged in sheets. In multiple areas, the subcutis is also infiltrated. Hematoxylin and eosin (H&E) stain, 4×. (**b**) **Primary cutaneous epitheliotropic lymphoma (mycosis fungoides), dunnart 4.** Dermal infiltrate predominantly comprises lymphocytes with hyperchromatic, convoluted or cerebriform nuclei admixed with occasional histiocytes and granulocytes. Multifocally, hair follicles are effaced by the infiltration of low numbers of neoplastic cells within the outer root sheath. H&E, 40×. (**c**) **Primary cutaneous epitheliotropic lymphoma (mycosis fungoides), dunnart 5.** Multiple intraepidermal vesicles are filled with pleomorphic lymphoid cells (Pautrier’s microabscesses) and occasionally by solitary cells surrounded by a clear halo. H&E, 40×. (**d**) **Peripheral diffuse cutaneous epitheliotropic lymphoma, dunnart 1.** Sheets of neoplastic lymphocytes are observed expanding the dermis, displacing adnexa and infiltrating and expanding the epidermis forming discrete and often coalescing Pautrier’s microabscesses. CD3 immunohistochemistry—IHC, 20×. (**e**) **Peripheral diffuse cutaneous epitheliotropic lymphoma, dunnart 1.** Neoplastic lymphocytes also reached into and isolated adipocytes. CD3 IHC, 10×.

In the first subset, significant non-reproductive disease in males was represented by three cases (dunnarts 1, 4, and 5) of primary cutaneous epitheliotropic lymphoma, resulting in severe and extensive gross cutaneous lesions (Figure 3d,e) compared to a lymphoplasmacytic dermatitis of unknown origin (Figure 3f). Telogen alopecia was also observed in four geriatric males (Figure 3g,h) occasionally along comedone formation (Figure 3i). In the second autopsied subset of dunnarts, two additional male dunnarts (dunnarts 31 and 33) were found with round cell infiltrates in the dermis, all manifesting in a clear sheet pattern but with dunnart 31 lacking epitheliotropism. On histopathology, the cutaneous epitheliotropic lymphoma cases presented with a densely cellular neoplasm arranged in sheets (Figure 5a) expanding the dermis, reaching the subcutis, and multifocally infiltrating the follicular epithelium (Figure 5b) extending to the epidermis filling vesicles (Figure 5c).

### 3.7. Histochemistry and Immunohistochemistry (IHC)

Toluidine blue (TB)—a metachromatic dye that stains cytoplasmic heparin granules in mast cells a deep purple color—was run in the second group of autopsied dunnarts that had cutaneous round cell infiltrates as there were frequently larger, rounded cells accompanying the primary infiltrating population. These larger cells corresponded to mast cells and their metachromatic granules were highlighted with TB. Regarding IHC controls, dunnart nodal tissue had adequate immunolabeling patterns of paracortical T (CD3) and cortical B cell (CD20, CD79a, Pax-5) markers, and IHC recovery was similar in all samples. However, there was minimal pan-leukocytic (CD68), histiocytic (Iba-1) or hemopoietic tumor markers’ (CD45 LCA) reactivity in control tissue. Pan-cytokeratin labeling was adequate in control tissue, and negative in the cells inducing the cutaneous round cell infiltrates. 

### 3.8. Non-Reproductive, Non-Clinically Significant Findings—Geriatric Male and Female Dunnarts

In the first subset, non-reproductive lesions were also observed but was generally considered of little or with no clinical significance (e.g., mild inflammatory infiltrates in stomach, intestine, kidneys, and/or liver—five cases combined; mild hepatocellular lipidosis—two cases; or mild hepatic glycogen storage—one case), associated with senile changes (e.g., multifocal mild renal mineralization or fibrosis—three cases; hepatic lipofuscinosis—one case) or euthanasia artefacts (e.g., pulmonary hemorrhages, congestion and edema—seven cases).

## 4. Discussion

In captivity, female Julia Creek dunnarts become sexually mature between 17 and 27 weeks and males between 28 and 31 weeks [4]. Similar to other dasyurids, dunnarts have evolved reproductive strategies to maximize fitness within their short lifespans (2–3 years), such as being seasonally polyestrous, an avoidance of semelparity (rapid senescence and death after first breeding season as in *Antechinus* sp.), having a spontaneous ovulation, multiparity and the ability to store sperm, which are unique amongst marsupials [12,13]. In addition, in situ surveys have also found that Julia Creek dunnarts can raise two litters in an extended breeding season, which is a common reproductive trait in species inhabiting semi-arid ecosystems where food availability is unpredictable [4]. Despite these adaptations, captive dunnarts unable to breed rapidly attain senescence, increasing their vulnerability to aging-associated conditions as observed in this study. Photoperiod cues could offer some insight into reproductive failure, particularly in male dasyurids. As photoperiod increases in the southern hemisphere, plasma cortisol levels do so as well, negatively influencing spermatogenesis [14]. Mortality coincides in certain dasyurids with elevated post-mating plasma cortisol levels as well as a testosterone-dependent, post-mating decrease in plasma corticosteroid-binding globulin. Such reproductive strategy might bring positive, short-term reproductive benefits for species evolved to have few mating encounters, via increasing energy mobilization. However, it can also induce fatal syndromes resulting in anemia, anorexia, hippocampal neuronal apoptosis, increased vulnerability to parasitic and bacterial infections, and decreased splenic follicle size, amongst other issues. The role of reproduction in post-mating morbidity and mortality is further supported by the significant increase in lifespan upon castration or pre-mating removal of males [14].

Susceptibility to age- and non-age-related hyperplasias, benign and malignant tumors vary considerably between marsupials or dasyurids. Interestingly and according to the literature, dasyurids are overrepresented in terms of neoplastic disease compared to other mammals, with one study reporting an incidence of 46% in this family [10] compared to 2–10% reported for other mammals [15]. None of the reported tumors of high incidence in dasyurids or other marsupials such as adenocarcinomas, mammary carcinomas, trichoepitheliomas, squamous cell carcinomas, hemangiomas or hemangiosarcomas, and hepatic adenomas were observed in this study, with the exception of lymphoma [14,16] and one case of a fibrosarcoma in a hind limb (not included in this study). Neoplasia in Julia Creek dunnarts was only represented by multiple cutaneous lymphomas and a uterine adenoma. Additionally, the literature reports degenerative conditions in geriatric dasyurids primarily affecting nervous or musculoskeletal system. Aged quolls (*Dasyurus* sp.) and Tasmanian devils (*Sarcophilus harrisii*) (>3 years old) acquire progressive leukoencephalopathies, myelopathies and intervertebral disc disease [17]; these were not observed in the dunnarts in this study.

It is important to note that the present study had a relatively small sample size and unfortunately not all dunnarts received a thorough postmortem examination with histopathology. These factors could have negatively influenced our ability to have a more accurate demonstration of geriatric dunnart diseases. Nonetheless, the literature on dunnart diseases is very limited compared to other marsupials. This fact is not surprising as diseases are not considered a significant threat to dunnart conservation, compared to other dasyurids with well-studied diseases such as the case with Tasmanian devils [18], or other marsupials, such as koalas (*Phascolarctos cinereus*) [19]. This fact has understandably re-directed research efforts into mapping conservation-priority habitat or answering ecology or biology questions [4,7,12,20]. A few studies regarding naturally occurring disease in captive fat-tailed dunnarts (*S. crassicaudata*) documented dermal spindle cell tumors, splenic lymphomas, squamous cell carcinomas and round cell sarcomas of the upper limb [10], whilst others stressed the vulnerability of captive dunnarts to the reverse transmission of zoonotic pathogens when human-derived *Helicobacter pylori* infection resulted in an outbreak of gastric bleeding and weight loss [21]. The cause of the high incidence of neoplasia is currently unknown, but genetic predisposition to lymphoma has been suggested in fat-tailed dunnarts, which grants further exploration of this question in dunnart species [10]. 

A small amount of information is available in the peer-reviewed literature on ovarian tumors in marsupials, or more specifically, dasyurids, with scattered reports of ovarian hemangiomas and adenocarcinomas in quolls and koalas [22,23]. Most of the lesions found in the female reproductive tract of these dunnarts are considered degenerative changes, namely CEH and cystic oviduct, which, as in other species, are associated with long-term exposure to female reproductive hormones in non-castrated animals [24]. Interestingly, this prolonged exposure is also a risk factor for mammary neoplasia, which was not observed in this study despite being reported in other marsupials [16,25]. The reasons behind this discrepancy are unknown. It is worth noting that in our study, 5 of 11 female dunnarts that did not meet the inclusion criteria (autopsy at NWT&R with no histopathology) also had gross ovarian lesions (dunnarts 134, 159, 170, 200), illustrating the high frequency of reproductive lesions in this colony. 

As reported elsewhere in possums [16,23], CGH was the most common disorder of growth in aged female dunnarts, as in aged rabbits, mice, rats and dogs [26,27,28]. Uterine cysts and uterine metaplasia have also been reported in mature gray short-tailed possums (*Monodelphis domestica*) [29]. Malignant neoplasia appears to be of common occurrence in the marsupial literature [10,30], and from these, carcinomas occur but are more often associated with other tissues (e.g., pulmonary, mammary gland) rather than endometrium [16,30,31,32,33]. Interestingly, primary malignant reproductive neoplasia was not observed in this study, and likewise, it was found to not be the major cause of death in a captive colony of endangered mountain pygmy-possum (*Burramys parvus*), with the report focusing instead on progressive renal disease [25]. Endometrial carcinomas are uncommon in animals except for the rabbit, cattle and certain rat strains. Multiple parallels regarding reproduction have been identified between rabbits and dunnarts which might provide insights into the pathogenesis of reproductive disorders in dunnarts. The two most common uterine disorders in rabbits are adenocarcinomas—affecting rabbits at any age and commonly resulting in pulmonary metastases—and CGH, with endometrial inflammation being uncommon [34,35]. CGH was the only entity frequently observed in this study. Notably, vaginal serosanguineous discharge and abdominal distension were frequent clinical findings in captive Julia Creek dunnarts with CGH and are also observed in rabbits with adenocarcinoma [35,36]; thus, it is recommended that any future captive breeding attempt in the species incorporates frequent checkups for either of these two clinical signs, with culling being a suitable endpoint. Ovariohysterectomy is the only successful treatment to date, with an overall post-surgical survival of 80% in rabbits [35]; however, this would not be a viable treatment option for a captive breeding colony, where fertility is important. In rabbits with endometrial adenocarcinoma, the non-carcinomatous foci in the endometrium have evidence of CGH, suggesting an association with hyperestrogenism. As induced ovulators, both rabbits and dunnarts can have quite prolonged estrus and experience little luteal activity, resulting in a persisting estrogen/progesterone ratio favoring estrogens [37]. At least in the rabbit, there is evidence of CGH evolving to malignant anaplastic carcinoma [36], so it is unknown if given sufficient time, the CGH dunnart cases would have turned malignant. Future research in this area evaluating the degree of hormone dependence of endometrial carcinomas and CGH could help in understanding the pathogenesis of spontaneous proliferative reproductive disorders in dunnarts. 

Both cases of neutrophilic infiltration in cervix and vagina could suggest an acute ascending bacterial infection—despite bacteria not observed on histopathology—or alternatively, their presence is possibly contingent to the phase of the ovarian cycle. Vaginal and cervical inflammation have been reported before in marsupials, mostly due to ascending bacterial infections from the cloaca (site of convergence for urogenital and alimentary tract) or due to tears during mating with an overly aggressive male [38]. Equally, the presence of granulocytes/neutrophils—along cornified and nucleated epithelial cells—in vaginal and cervical segments is a normal finding in mice and rats during metestrus and diestrus, serving as an arm of localized innate immunity and assisting when staging vaginal smears [39,40]. It is unknown to the authors if the dasyurid ovarian cycle behaves similarly to rodents. The literature on the cellular reproductive profile in other marsupials (e.g., Tasmanian rat-kangaroo (*Potorus tridmtylus*) [41] and Common brushtail possum (*Trichosurus vulpecula*)) [42] suggests that the ovarian cyclic phase might influence rodents and marsupials in a similar fashion. Nonetheless, it is worth noting that both female dunnarts in which neutrophilic infiltration was observed had concurrent CGH and evidence of anovulatory ovaries, which may favor a pathologic cause for neutrophilic recruitment. 

Cases of testicular degeneration are consistent with advanced age as reported elsewhere in other aged male marsupials [29]. In one case (dunnart 9), the left testicle was unilaterally enlarged, necrotic and with a diffusely edematous tunica albuginea, suggesting that the necrosis was secondary to impaired thermoregulation [43]. Testicular atrophy is seen as an advanced state of degeneration, with mineralization and fibrosis. Causes are multiple; however, intrinsically, aging is a well-recognized predisposing factor in all species, probably due to degenerative vascular lesions in testis or pampiniform plexus [43]. Aggregates of interstitial (Leydig) cells were observed in one case of testicular degeneration with concurrent seminiferous tubular loss and replacement by granulomas within a background of autolysis. It is possible that the loss of tubular profiles mimicked interstitial cell hyperplasia, a recognized phenomenon in aged animals with or without atrophied testes. If the cellular proliferation was a real change, hyperplasia would have been the more likely interpretation instead of adenoma, as the diameter of the proliferating foci was less than three seminiferous tubules [44]. It is also worth noting that testicular interstitial cells are prominent in marsupials, particularly in American marsupial families (and Australian bandicoots), where the proportion of testicular volume occupied by the interstitial cells can reach up to 20%, known as a type 3 organization [45]. Although most Australian marsupials are documented to have a type 2 organization, occupying about 5% of testicular volume [45], some dasyurids such as *Antechinus stuartii* are known to have two separate interstitial cell populations, with one forming peritubular clusters thought to correspond to renewed cells in adulthood [46]. It is unknown if this characteristic is shared by all dasyurid males [47], but once again, such peritubular clustering could have mimicked hyperplasia. Although the functional significance and phylogenetic connotations of such changes are not unknown, familiarity of the anatomic pathologist with such anatomical variations is of utmost importance to perform adequate histological examinations in non-conventional species. 

Prostatitis has been reported in sugar gliders (*Petaurus breviceps*) and Virginia opossums (*Didelphis virginiana*), mostly associated with urinary tract infection, resulting in hematuria, constipation and local pain; none of which were observed in this study perhaps due to the mild nature of the inflammation observed in dunnart 11 [31,38]. Sexually-transmitted, cytomegalovirus-associated prostatitis has been reported in other Australian dasyurids, the brush-tailed phascogale (*Phascogale tapoatafa*), the brown antechinus (*Antechinus stuartii*) and the dusky antechinus (*A. swainsonii*; [48,49]). As with other cytomegaloviruses, lesions were most frequent during stressful periods. If the inflammation observed in dunnart 11 was virally induced, it is unlikely that viable virions would still be observable in the tissue due to chronicity. Nonetheless, future studies should consider a potential cytomegalovirus infection in dasyurid cases showing prostatic compromise. Prostatic mineralization is considered a senile change lacking clinical significance.

Cutaneous lymphoma was frequent in male dunnarts. There were two females in which both gross and histopathology findings could support a putative diagnosis of lymphoma; however, this diagnosis could not be confirmed due to the severe artefact present in the tissue, impeding confirmatory testing. Thus, we will only discuss cutaneous epitheliotropic lymphoma in the male cohort. Dunnarts with cutaneous epitheliotropic lymphomas presented with extensive alopecia with crusting and ulcerative lesions in the skin. Interestingly, lesions were remarkably similar in distribution and severity, which could indicate a viral etiology. Oncogenic viruses, specifically those of the retroviral lineage, such as the Koala Retrovirus and Gunnison’s Prairie Dog Retrovirus are associated with lymphoid neoplasia in wildlife in Australia and elsewhere [50,51]. This hypothesis might be worth exploring in future studies. Cutaneous epitheliotropic T-cell lymphoma (ETL) is primarily a disease of aged animals and has been reported in a variety of marsupials [52,53] and dasyurids, including Tasmanian devils [54]. In dogs, lesions develop from a patch displaying significant epitheliotropism into a tumor stage with metastasis in as little time as 5.5 months [55], with cutaneous forms (as the dunnarts in this study) considered independent predictors of poor survival compared to mucocutaneous/mucosal forms [56]. The ETL disease spectrum is divided into mycosis fungoides (MF; most common form, strong tropism for epidermis and adnexa), pagetoid reticulosis (MF with lymphadenopathy and circulating neoplastic lymphocytes) and Sézary syndrome (solitary plaque; [57]). According to this classification, the three ETL cases in this study correspond to MF due to the presence of (1) intraepidermal vesicles containing pleomorphic neoplastic lymphocytes (Pautrier’s microabscesses), (2) infiltration of follicles and adnexa (epitheliotropism), and (3) pleomorphic infiltrate predominantly comprising lymphocytes with occasional histiocytes and granulocytes. Predisposing factors to cutaneous ETL are not known; however, chronic inflammation and immune dysregulation, as it occurs in atopic dermatitis cases, is noted as a risk factor for dogs [58]. It remains to be determined if chronic dermatitis, as diagnosed in one male excluded from the study, predisposes aged dunnarts to cutaneous ETL. Although speculative, another possible risk factor for dunnart lymphoma occurrence is the male sex, as indicated by previous studies in neutered dogs, and humans [59,60], suggesting an endocrine dysregulation component. Interestingly and perhaps unsurprisingly, two of the three dunnarts with ETL also had concurrent testicular degeneration, which would lead to low circulating testosterone levels and, subsequently, supraphysiologic luteinizing hormone (LH) levels due to the absence of negative feedback to the hypothalamus and anterior pituitary axis, as would happen in gonadectomized animals. LH has receptors in reproductive and non-reproductive tissues such as lymphocytes and lymphoid tissue (thymic medulla); the function of these receptors is still not fully understood but appears to relate to the induction of cell function and division [61], with the latter via an ERK-dependent pathway [62]. High LH would lead to a constant activation and magnification of LH effects in non-reproductive tissues, including T lymphocytes [63]. LH-receptor positive T lymphocytes have been shown to circulate in higher numbers in gonadectomized dogs compared to intact individuals [64], plausibly stimulating cell division through T-lymphocyte-bound LH receptor activation. These findings correspond to outcomes of in vitro experiments and thus, prospective case–control trials are required to further verify a causative association [63,65]. Nonetheless, if dunnarts with testicular degeneration have comparable endocrine profiles to gonadectomized dogs, it could potentially lead to similar pathophysiological effects of sustained supraphysiologic LH levels. It is also unknown, however, if the onset of testicular degeneration in the male dunnarts included in this study occurred earlier or later in life. We speculate testicular degeneration is likely to be a consequence of aging, as its occurrence in younger or pre-pubertal dunnarts would likely have led to other significant health issues due to the loss of negative feedback to the hypothalamus and anterior pituitary. In dogs and humans, sustained supraphysiologic levels of LH have been documented to lead to metabolic (hypothyroidism), musculoskeletal issues (increased ligament laxity) or urinary issues (incontinence) [63,65]. Lesions compatible with these health issues were not observed in the dunnarts included in this study. 

The insufficient leukocytic marker immunolabeling observed in nodal control tissue hampered our ability to utilize IHC to definitively immunophenotype the cutaneous lymphomas observed. Possible issues that could have negatively impacted IHC immunolabeling quality include poor preservation of sample prior to freezing, age of samples (nearly a decade old), preservation artefact (freezing), lack of proper validation procedures in the species and prolonged or variable formalin fixation time between samples [66]. Pan-cytokeratin did provide adequate diagnostic immunolabeling in control tissue, facing the same preservation and fixation challenges. However, with pan-cytokeratin being a different epitope, it is unlikely to be equally negatively influenced by the aforementioned challenges that did affect leukocytic and hematopoietic markers. 

Hair loss has been extensively documented in captive wildlife and can be caused by stress, excessive grooming, parasites, integument diseases, dietary deficiencies and infections (fungal and bacterial), amongst others. The alopecia observed could have some similarity with telogen effluvium, a well-described condition in domestic animals in which there is pelage loss associated with extrinsic physical, physiological or mental stressors or improper diets. With telogen effluvium, there is excessive generalized hair shedding with a reduction in hair volume that does not render complete baldness. The pattern of alopecia observed in the three males affected were similar to what has been reported in macaques [67] and bats [68], where stress has been detected as an important cause. Further, comedone formation, although infrequently observed, could support underlying stress or endocrinopathies as it has been documented in small animals [69].

Barbering—the act of plucking whiskers and fur in mice due to excessive grooming—would be an important differential diagnosis in these cases if it is confirmed that captive dunnarts can replicate such behavior responding to social dominance [70]. Excessive hair pulling (trichotillomania) is different and has also been observed in macaques and can be partially corrected with environmental enrichment [71]. Despite whiskers being intact in this study, males were over-represented with alopecia, which may grant the exploration of hierarchical conflicts with females as a cause, as female mice are more likely to apply this behavior than males. As chronic stress is a well-recognized issue in captive wildlife and has been found to be associated with conditions observed in this study, future attempts at establishing captive breeding dunnart colonies could benefit from exploring environmental enrichment as well as the incorporation of non-invasive fecal glucocorticoid metabolite testing or hair cortisol levels as biomarkers to monitor stress levels [72]. 

Senescence is a risk factor for cancer development. Both mature age and cancer share disease mechanisms such as genomic instability, epigenetic changes, altered nutrient sensing metabolism and are fundamentally divergent in others, such as telomere attrition in geriatric cells in contrast to the ability to perform rapid cell division and high energy consumption of neoplastic cells [73]. The lifespan of captive Julia Creek dunnarts was increased by approximately one year, and so, the molecular mechanisms associated with aging are suggested as the underlying mechanisms for the interconnectedness of aging and neoplasia observed in this study. Nonetheless, future studies should aim at investigating other potential etiologies including oncogenic viruses. 

Reproductive aging is a crucial phenomenon of concern in any conservation breeding program, particularly for genetically valuable wildlife, as research has shown long-term captivity can lead to prolonged exposure to endogenous sex steroids and long non-reproductive intervals, resulting in asymmetric reproductive aging and low reproductive performance [74]. In asymmetric reproductive aging, females in which first breeding attempts and pregnancies are substantially delayed result in frequent cyclic fluctuations of estrogen, subsequently causing reproductive disease, as tested in cheetahs (*Acinonyx jubatus*) [75]. The theory is interesting and could be applied to dasyurids, although its veracity remains to be tested. To avoid asymmetric reproductive aging, reduced fertility and irreversible acyclicity, especially in short-lived wildlife, captive breeding programs can benefit from incorporating a basic cytological assessment of reproductive cycle phases (e.g., urogenital/vaginal mucus or urine smears [76,77,78,79,80] to monitor fertility and perform early and regular selective breeding of young and genetically valued females). Alternatively, captive breeding programs could benefit from the use of pioneered assisted reproductive technologies to induce pregnancies as early as possible [74,81,82], or monitoring for behavioral signals (e.g., wheel running activity [83]). 

## 5. Conclusions

From the findings reported in this study, it can be inferred that captive breeding colonies of dunnarts can benefit from including health examinations into their program, particularly upon reaching geriatric age. This study is the first to document a comprehensive list of spontaneous reproductive and significant non-reproductive conditions affecting geriatric dunnarts to improve the paucity of the dasyurid disease literature. These results paired with effective management prescriptions, including captive breeding, maintenance and restoration of land with essential structure for the species, are vital for the long-term conservation success of Julia Creek dunnarts. 

## Figures and Tables

**Table 1:** Female reproductive and non-reproductive tract lesions in endangered geriatric Julia Creek dunnarts from a captive breeding colony.

Diagnoses	Dunnart ID	Age (Months)
Ovaries		
Cystic ovary (possibly bursal)	6	25
Age-related ovarian atrophy	8	25
Age-related ovarian atrophy	10	42
Oviduct		
Cystic oviduct	10	42
Uterus		
Cystic glandular hyperplasia, squamous metaplasia	3	27
Cystic glandular hyperplasia/dysplasia, squamous metaplasia	7	25
Cystic glandular hyperplasia with mucin	8	25
Cystic glandular hyperplasia	2	24
Cystic glandular hyperplasia	10	42
Cystic glandular hyperplasia	24	24
Cystic glandular hyperplasia, polyp (glandular)	27	24
Cystic glandular hyperplasia, squamous metaplasia	11	20
Cystic glandular hyperplasia, squamous metaplasia, polyp (glandular)	14	42
Endometrial polyp (glandular)	23	12
Endometrial polyp (glandular), cystic	30	24
Endometrial polyp (glandular), cystic	32	24
Endometrial adenoma, papillary, focal squamous metaplasia	13	42
Cervix and vagina		
Neutrophilic infiltrates in cervix and vagina with fibrosis	8	25
Neutrophilic infiltrates in vagina	10	42
Skin		
Round cell infiltrates, superficial dermis, non-epitheliotropic	30	24
Round cell infiltrates, mid-to-deep dermal, epitheliotropic	33	12

**Table 2:** Male reproductive and non-reproductive tract lesions in endangered geriatric Julia Creek dunnarts from a captive breeding colony.

Diagnoses	Dunnart ID	Age (Months)
Testes		
Testicular tubular degeneration/atrophy with aspermatogenesis	4	42
Testicular tubular degeneration/atrophy with aspermatogenesis and metastatic lymphoma	5	42
Testicular tubular degeneration/atrophy	9	42
Prostate		
Prostatic mineralization, multifocal	9	42
Lymphoplasmacytic prostatitis	12	42
Skin		
Cutaneous epitheliotropic lymphoma (mycosis fungoides)	1	24
Cutaneous epitheliotropic lymphoma (mycosis fungoides), with splenic and pulmonary metastasis	4	42
Cutaneous epitheliotropic lymphoma (mycosis fungoides), with splenic, pulmonary and testicular metastasis	5	42
Cutaneous round cell infiltrates, telogen alopecia	25	48
Cutaneous round cell infiltrates	31	48
Telogen alopecia, hyperkeratosis	20	36
Telogen alopecia	28	24
Telogen alopecia	29	24

## Data Availability

The data presented in this study are available on request from the corresponding author. The data are not publicly available due to inclusion of personal data from submitters.

## Supplementary Materials

The following supporting information can be downloaded at: https://www.mdpi.com/article/10.3390/vetsci11040142/s1, Table S1: Pathology examination and patient outcomes of Julia Creek dunnart colony at The University of Queensland, School of Veterinary Science; Figure S1: Investigation into the immunolabeling of CD3 (membranous to cytoplasmic) leukocytic marker in Julia Creek dunnart tissue. (A) Female dunnart 33 (second batch). (A1) Lymph node. Normal distribution of target antigen is observed within the nodal paracortex, where the chromogen has a membranous, perinuclear or cytoplasmic reactivity within paracortical leukocytes, especially T-lymphocytes mimicking the distribution of the internal laboratory control (human tonsil). (A2) Lymph node. Higher magnification image of a cortico-follicular-paracortical region of a second lymph node, demonstrating proper membranous, perinuclear to cytoplasmic immunolabeling, indicating the presence of T-lymphocytes. (A3) Haired skin. The dermis is heavily infiltrated by an unidentified round cell population that is negative to the target antigen. All epidermal layers, dermal fibroblasts and collagen are negative (internal controls). Faint, cytoplasmic immunolabeling is observed within larger rounded cells scattered in the dermis—possibly local unidentified lymphoid cells such as NK cells (inset). (A4) Ovary. The ovarian stroma is diffusely negative to the target antigen. Non-specific, faint, random extracellular immunolabeling is observed within ovarian follicles. (B) Male dunnart 5 (first batch). (B1) Haired skin. Diffuse, strong, membranous, perinuclear to cytoplasmic immunolabeling is observed in all round cells infiltrating and expanding the entire dermis, abutting the panniculus adiposus. No immunolabeling is observed in adipocytes or skeletal myocytes (internal negative controls). (B2) Haired skin. Higher magnification of B1. The sheets of positive, moderate to strong immunolabeled round cells are multifocally interrupted by larger, rounded, strongly immunolabeled cells (possible local unidentified lymphoid cells, such as NK cells). (B3) Intestine. Gut-associated lymphoid tissue (GALT) T-cells demonstrate membranous, cytoplasmic reactivity. (B4) Haired skin. High magnification of B3. Matrical melanocytes display strong immunoreactivity to the target antigen. Follicular keratinocytes and adipocytes are negative to the target antigen (internal negative controls). (C) Male dunnart 31 (second batch). (C1) Prostate. Random, moderate to strong membranous, perinuclear to cytoplasmic immunolabeling of leukocytes (possibly tissue macrophages) located in the inter-glandular stroma. Faint to light, random, extracellular and intracellular—non-specific staining is observed within the apical portion of columnar cells and glandular lumina (background staining). (C2) Prostate. High magnification image of C1. (C3) Haired skin. Similar to Dunnart 33, the skin contains a marked infiltration by round cells arranged in sheets; all negative to the CD3 leukocytic marker. A scattered population of larger, rounded cells contain cytoplasmic immunolabeling with CD3 (possibly tissue macrophages). (C4) Haired skin. Higher magnification of C3. No immunolabeling is observed within keratinocytes (upper right corner) or dermal fibroblasts and collagen. Minimal cytoplasmic labeling is observed within mast cells (confirmed via toluidine blue). Control tissue: Canine tonsil. Tonsillar tissue of both epithelial cell lineages and lymphatic lineages, serving as positive control for lymphatic markers and negative control for lymphatic markers. Tumors are avoided due to the variable expression of markers. Figure S2. Investigation into the immunolabeling of CD20 (membranous) leukocytic marker in Julia Creek dunnart tissue. All tissues belong to dunnart 33 (second batch). (A) Female dunnart 33 (second batch). (A1) Lymph node. Follicular structures show reactivity, evenly spaced out from a lymphoid population lacking reactivity, likely corresponding to paracortical T-cells. (A2) Lymph node. Higher magnification of A1. Focal membranous to cytoplasmic reactivity is observed within a lymphoid follicle, likely detecting pre-plasma cell differentiation, mature B cells and possibly follicular dendritic cells. (A3) Lymph node. Higher magnification of A2. (A4) Uterus. The myometrium shows a lack of reactivity, except for faint, random, non-specific reactivity of smooth muscle myocytes within stratum vasculare. (A5) Haired skin. The dermis is markedly expanded by an infiltrating population of round cells forming sheets that shows a diffuse lack of reactivity. (A6) Haired skin. Higher magnification view of an upper epidermis expanded with round cells arranged in sheets. The dermal collagen, fibroblasts, epidermis and overlying serocellular crust covering an eroded epidermis show a lack of reactivity. (A7) Oviduct (isthmus). There is diffuse lack of reactivity of the smooth muscle myocytes and epithelium. (A8) Ovary. There is diffuse lack of reactivity in the stroma and follicular epithelium. Faint, non-specific, extracellular reactivity is observed infrequently within ovarian follicles. Control tissue: Canine tonsil and dunnart 33 lymph node. Canine tonsil tissue was added although CD20 has not been validated in this species, hence the lack of reactivity. Figure S3. Investigation into the immunolabeling of CD45 (membranous and cytoplasmic) leukocyte common antigen (LCA) marker in Julia Creek dunnart tissue. (A) Female dunnart 33 (second batch). (A1) Lymph nodes. Subgross view of cortex and medulla of two lymph node profiles with internodal stroma. Most of the cortex, all the paracortex, subcapsular and medullary sinuses and internodal stroma show a diffuse lack of reactivity. Within the cortex, a few scattered follicles have small numbers of reactive cells. (A2) Lymph nodes. Higher magnification image of cortical and paracortical tissue of the lymph node in A1 (upper right corner). (A3) Lymph nodes. Higher magnification image of cortical and paracortical tissue of lymph node in A1 (bottom). Reactivity is observed within the membrane and cytoplasm of rounded cells, which could correspond to nucleated cells of haemopoietic origin (lymphocytes, monocytes, macrophages). (A4) Lymph nodes. Higher magnification image of cortical and paracortical tissue of lymph node in A1 (bottom). Control tissue: Dunnart 33 lymph node. Figure S4. Investigation into the immunolabeling of CD68 (cytoplasmic) leukocytic marker in Julia Creek dunnart tissue. (A) Female dunnart 33 (second batch). (A1) Lymph node. Small clusters of reactive rounded cells are observed within cortical nodal tissue. The typical reaction pattern of dot-like granular or diffuse cytoplasmic reactivity is observed. (A2) Haired skin. The round cell population expanding the dermis shows a diffuse lack of reactivity. (A3) Haired skin. Higher magnification view of the epidermis, dermal collagen, fibroblasts, and follicular epithelium with a diffuse lack of reactivity. (A4) Uterus. The myometrium displays a diffuse lack of reactivity (internal control). Control tissue: Canine tonsil and dunnart 33 lymph node. Canine tonsil tissue was added although CD68 has not been validated in this species, hence the lack of reactivity. Figure S5. Investigation into the immunolabeling of CD79a (cytoplasmic or membranous) leukocytic marker in Julia Creek dunnart tissue. (A) Female dunnart 33 (second batch). (A1) Lymph node. Two cortical lymphoid follicles display small clusters of reactive cells. (A2) Lymph node. Higher magnification image containing a small cluster of cells displaying cytoplasmic reactivity is observed within a nodal cortical follicle, possibly identifying some pre-pro B cell or plasma cells. (A3) Lymph node. Higher magnification image nothing reactivity is predominantly cytoplasmatic. (A4) Medium caliber vessel. There is non-specific, moderate reactivity within smooth muscle myocytic sarcoplasm and extracellularly within the vascular lumen. Control tissue: Canine tonsil and dunnart 33 lymph node. Figure S6. Investigation into the immunolabeling of PAX5 (nuclear) leukocytic marker in Julia Creek dunnart tissue. (A) Male dunnart 28 (second batch). (A1) Pancreas. There is diffuse lack of nuclear reactivity within exocrine and endocrine pancreatic tissue. Epithelium lining a pancreatic ducts also lacks reactivity (top right corner). (A2) Esophagus. The parakeratinized epithelium lacks reactivity. Faint, non-specific, extracellular immunolabeling is observed in the esophageal lumen. (A3) Skeletal muscle. There is diffuse lack of nuclear reactivity within myocyte nuclei and satellite cells. A4) Lymph node. Scattered, non-specific, reactivity is observed within perinodal tissue. Control tissue: Canine tonsil. (B) Male dunnart 5 (first batch). (B1) Lymph node. High magnification image of a single lymphoid follicle displaying strong nuclear reactivity, likely identifying pre-pro-B cell through mature B cells. (B2) Brain. High magnification image containing a section of grey matter with hippocampal neurons, all lacking reactivity. (B3) Kidney. High magnification image containing a section of renal cortex with tubules and glomeruli lacking reactivity. (B4) Haired skin. The dermis is expanded by round cells arranged in sheets. A small serocellular crust is observed overlying a focally eroded epidermis. No nuclear reactivity is observed in any cell population. Control tissue: Dunnart 5 lymph node. (C) Female dunnart 33 (second batch). (C1) Lymph node. Subgross view of a second lymph node, in which small, scattered clusters of reactivity are observed. (C2) Haired skin. The dermis is infiltrated by round cells that lack nuclear reactivity. Dermal fibroblasts, collagen, follicular and epidermal epithelium also lack nuclear reactivity (internal negative control). (C3) Kidney. In the renal cortex, glomeruli and tubular epithelium lack nuclear reactivity (internal negative control). (C4) Lymph node. Higher magnification image of C2. Control tissue: Dunnart 33 lymph node. Figure S7. Investigation into the immunolabeling of Iba-1 (cytoplasmic) histiocytic marker in Julia Creek dunnart tissue. (A) Female dunnart 33 (second batch). (A1) Lymph node. Low magnification image of control lymph node where clusters of cells displaying cytoplasmic reactivity are observed within multiple lymphoid follicles. (A2) Lymph node. High magnification image of control lymph node on A1. (A2) Haired skin. Diffuse lack of reactivity is observed in a round cell population infiltrating and markedly expanding the dermis. (A3) Uterus. Smooth muscle myocytes lack cytoplasmic reactivity. Faint, multifocal, non-specific, extracellular reactivity is observed within the *stratum vasculare*. Control tissue: Canine tonsil and canine brain. Figure S8. Investigation into the immunolabeling of cytokeratin AE1/AE3 (cytoplasmic) marker in Julia Creek dunnart tissue. (A1) Lymph node. Scattered cytoplasmic reactivity is observed in small groups of cells centering lymphoid follicles. Outer follicular cells and paracortical cells diffusely lack reactivity. (A2) Lymph node. High magnification view of A1. (A3) Haired skin. Moderate to strong cytoplasmic reactivity is observed within follicular and epidermal keratinocytes. (A4) Haired skin. High magnification view of the epidermis with moderate cytoplasmic reactivity within keratinocytes, and diffuse lack of cytoplasmic reactivity in the round cell population infiltrating and expanding the dermis. Control tissue: Canine tonsil.
