# Supplementary material for: Spontaneous Lesions of Endangered Geriatric Julia Creek Dunnarts (Sminthopsis douglasi, Archer 1979) with Emphasis in Reproductive Pathology
Source: Vet Sci. 2024 Mar 22;11(4):142. doi: 10.3390/vetsci11040142 (PMC11054575; doi:10.3390/vetsci11040142)
Supplement: Supplementary file 1 [file vetsci-11-00142-s001.zip › Suppl Mat 1 VGA 22Jan24.pdf]

Supplementary Material 1. Immunohistochemistry protocols for common leukocytic and cellular markers applied to Julia Creek dunnart (*Sminthopsis douglasi*) tissue.

Immunohistochemistry was ran in lymph node tissue as control tissue for leukocytic markers (CD3, CD20, CD79a), pan-leukocytic (CD68), histiocytic (Iba-1), and haemopoietic tumor markers (CD45 LCA) and in skin and intestine samples to test pan-cytokeratin (AE1/AE3) labelling in 6 dunnarts (dunnarts 20, 25, 30-33) belonging to the second subset of autopsies. IHC was then carried out in skin samples from individuals of both subsets showing cutaneous round cell infiltrates. IHC was performed using DAKO OMNIS Automatic Stainer with 4-um sections and 30-minute heat fixation for tissue adhesion on DAKO IHC coated slides at 70°C. All antibody concentrations/titrations were available as commercial pre-dilutions (Agilent ®, Santa Clara, CA, USA). Variations to the protocol for each IHC marker (CD3, CD20, CD79a and PAX5) were carried out to follow internal validation protocols for quality control and institutional standard operating procedures utilizing tonsil tissue for positive and known negative cell populations. Histochemistry (Gram, Toluidine blue) was performed in a case-by-case basis following UQ's standard laboratory protocols.

<u>CD3</u>

1. Clearify 1min
2. Clearify 1min
3. DI Water Wash 5 sec
4. 95C High pH Heat Induced Epitope Retrieval (HIER) 30 min
5. Wash Buffer 3 min
6. CD3 Antibody 20 min
7. Wash Butter 2 min
8. Peroxidase Blocking Reagent 3 min
9. Wash Buffer 2 min
10. Rabbit Linker 10 min
11. Wash Buffer 2 min
12. Horse Radish Peroxidase (HRP) 20 min
13. Wash Buffer 2 min
14. Wash Buffer 2 min
15. DI Water 30 sec
16. Wash Buffer 2 min
17. DAB Working Sol. 5 min
18. Wash Buffer 2 min
19. DI Water 30 sec
20. Wash Buffer 2 min
21. Haematoxylin 3 min
22. DI Water 2 min
23. Wash Buffer 2 min
24. Dehydrate, Clear and Mount.

<u>CD20</u>

1. Clearify 1min
2. Clearify 1min
3. DI Water Wash 5 sec
4. 95C High pH Heat Induced Epitope Retrieval (HIER) 30 min
5. Wash Buffer 3 min
6. CD20 Antibody 15 min
7. Wash Butter 2 min
8. Peroxidase Blocking Reagent 3 min
9. Wash Buffer 2 min
10. Horse Radish Peroxidase (HRP) 20 min
11. Wash Buffer 2 min
12. Wash Buffer 2 min
13. DI Water 30 sec
14. Wash Buffer 2 min
15. DAB Working Sol. 5 min
16. Wash Buffer 2 min
17. DI Water 30 sec
18. Wash Buffer 2 min
19. Haematoxylin 3 min
20. DI Water 2 min
21. Wash Buffer 2 min
22. Dehydrate, Clear and Mount.

CD79a

1. Clearify 1min
2. Clearify 1min
3. DI Water Wash 5 sec
4. 95C High pH Heat Induced Epitope Retrieval (HIER) 30 min
5. Wash Buffer 3 min
6. CD79a Antibody (1:50 dilution) 30 min
7. Wash Butter 2 min
8. Peroxidase Blocking Reagent 3 min
9. Wash Buffer 2 min
10. Mouse Linker 10 min
11. Wash Buffer 2 min
12. Horse Radish Peroxidase (HRP) 20 min
13. Wash Buffer 2 min
14. Wash Buffer 2 min
15. DI Water 30 sec
16. Wash Buffer 2 min
17. DAB Working Sol. 5 min
18. Wash Buffer 2 min
19. DI Water 30 sec
20. Wash Buffer 2 min
21. Haematoxylin 3 min
22. DI Water 2 min
23. Wash Buffer 2 min
24. Dehydrate, Clear and Mount.

PAX5

1. Clearify 1min
2. Clearify 1min
3. DI Water Wash 5 sec
4. 95C High pH Heat Induced Epitope Retrieval (HIER) 30 min
5. Wash Buffer 3 min
6. PAX5 Antibody 15 min
7. Wash Butter 2 min
8. Peroxidase Blocking Reagent 3 min
9. Wash Buffer 2 min
10. Mouse Linker 10 min
11. Wash Buffer 2 min
12. Horse Radish Peroxidase (HRP) 20 min
13. Wash Buffer 2 min
14. Wash Buffer 2 min
15. DI Water 30 sec
16. Wash Buffer 2 min
17. DAB Working Sol. 5 min
18. Wash Buffer 2 min
19. DI Water 30 sec
20. Wash Buffer 2 min
21. Haematoxylin 3 min
22. DI Water 2 min
23. Wash Buffer 2 min
24. Dehydrate, Clear and Mount.

CD45 LCA

1. Clearify 1min
2. Clearify 1min
3. DI Water Wash 5 sec
4. 95C High pH Heat Induced Epitope Retrieval (HIER) 30 min
5. Wash Buffer 3 min
6. CD45 LCA Antibody 10 min
7. Wash Butter 2 min
8. Peroxidase Blocking Reagent 3 min
9. Wash Buffer 2 min
10. Rabbit Linker 10 min
11. Wash Buffer 2 min
12. Horse Radish Peroxidase (HRP) 15 min
13. Wash Buffer 2 min
14. Wash Buffer 2 min
15. DI Water 30 sec
16. Wash Buffer 2 min
17. DAB Working Sol. 5 min
18. Wash Buffer 2 min
19. DI Water 30 sec
20. Wash Buffer 2 min
21. Haematoxylin 3 min
22. DI Water 2 min
23. Wash Buffer 2 min
24. Dehydrate, Clear and Mount.

CD68

1. Clearify 1min
2. Clearify 1min
3. DI Water Wash 5 sec
4. 95C High pH Heat Induced Epitope Retrieval (HIER) 30 min
5. Wash Buffer 3 min
6. CD68 Antibody 20 min
7. Wash Butter 2 min
8. Peroxidase Blocking Reagent 3 min
9. Wash Buffer 2 min
10. Rabbit Linker 10 min
11. Wash Buffer 2 min
12. Horse Radish Peroxidase (HRP) 20 min
13. Wash Buffer 2 min
14. Wash Buffer 2 min
15. DI Water 30 sec
16. Wash Buffer 2 min
17. DAB Working Sol. 5 min
18. Wash Buffer 2 min
19. DI Water 30 sec
20. Wash Buffer 2 min
21. Haematoxylin 3 min
22. DI Water 2 min
23. Wash Buffer 2 min
24. Dehydrate, Clear and Mount.

IBA-1

1. Clearify 1min
2. Clearify 1min
3. DI Water Wash 5 sec
4. 95C Low pH Heat Induced Epitope Retrieval (HIER) 30 min
5. Wash Buffer 3 min
6. IBA-1 Working solution (1:300 dilution with antibody diluent from Agilent), antibody 20 min
7. Wash Butter 2 min
8. Peroxidase Blocking Reagent 3 min
9. Wash Buffer 2 min
10. Rabbit Linker 10 min
11. Wash Buffer 2 min
12. Horse Radish Peroxidase (HRP) 20 min
13. Wash Buffer 2 min
14. Wash Buffer 2 min
15. DI Water 30 sec
16. Wash Buffer 2 min
17. DAB Working Sol. 5 min
18. Wash Buffer 2 min
19. DI Water 30 sec
20. Wash Buffer 2 min
21. Haematoxylin 3 min
22. DI Water 2 min
23. Wash Buffer 2 min
24. Dehydrate, Clear and Mount.

AE1/AE3

1. Clearify 1min
2. Clearify 1min
3. DI Water Wash 5 sec
4. 95C High pH Heat Induced Epitope Retrieval (HIER) 30 min
5. Wash Buffer 3 min
6. AE1/AE3 Antibody 20 min
7. Wash Butter 2 min
8. Peroxidase Blocking Reagent 3 min
9. Wash Buffer 2 min
10. Rabbit Linker 10 min
11. Wash Buffer 2 min
12. Horse Radish Peroxidase (HRP) 20 min
13. Wash Buffer 2 min
14. Wash Buffer 2 min
15. DI Water 30 sec
16. Wash Buffer 2 min
17. DAB Working Sol. 5 min
18. Wash Buffer 2 min
19. DI Water 30 sec
20. Wash Buffer 2 min
21. Haematoxylin 3 min
22. DI Water 2 min
23. Wash Buffer 2 min
24. Dehydrate, Clear and Mount.
