# Supplementary material for: Spontaneous Lesions of Endangered Geriatric Julia Creek Dunnarts (Sminthopsis douglasi, Archer 1979) with Emphasis in Reproductive Pathology
Source: Vet Sci. 2024 Mar 22;11(4):142. doi: 10.3390/vetsci11040142 (PMC11054575; doi:10.3390/vetsci11040142)
Supplement: Supplementary file 1 [file vetsci-11-00142-s001.zip › Supplementary Figures IHC validation 1-8.pdf]

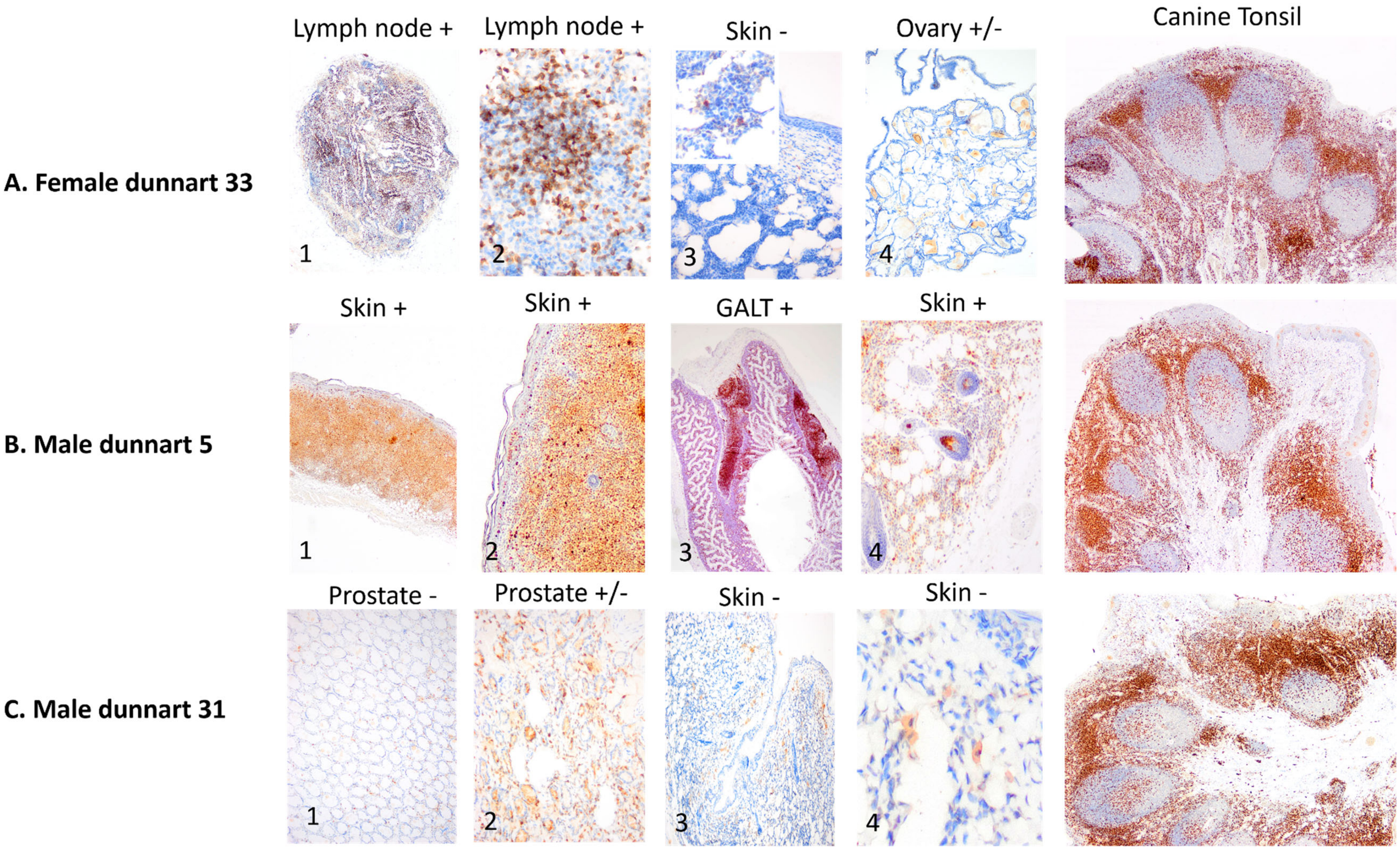

Supplementary Figure 1. Investigation into the immunolabeling of CD3 (membranous to cytoplasmic) leukocytic marker in Julia Creek dunnart tissue.

A) Female dunnart 33 (second batch). A1) Lymph node. Normal distribution of target antigen is observed within the nodal paracortex, where the chromogen has a membranous, perinuclear or cytoplasmic reactivity within paracortical leukocytes, especially T-lymphocytes mimicking the

distribution of the internal laboratory control (human tonsil). **A2) Lymph node.** Higher magnification image of a cortico-follicular-paracortical region of a second lymph node, demonstrating proper membranous, perinuclear to cytoplasmic immunolabeling, indicating the presence of T-lymphocytes. **A3) Haired skin.** The dermis is heavily infiltrated by an unidentified round cell population that is negative to the target antigen. All epidermal layers, dermal fibroblasts and collagen are negative (internal controls). Faint, cytoplasmic immunolabeling is observed within larger rounded cells scattered in the dermis – possibly local unidentified lymphoid cells such as NK cells (inset). **A4) Ovary.** The ovarian stroma is diffusely negative to the target antigen. Non-specific, faint, random extracellular immunolabeling is observed within ovarian follicles.

B) Male dunnart 5 (first batch). B1) Haired skin. Diffuse, strong, membranous, perinuclear to cytoplasmic immunolabeling is observed in all round cells infiltrating and expanding the entire dermis, abutting the panniculus adiposus. No immunolabeling is observed in adipocytes or skeletal myocytes (internal negative controls). **B2) Haired skin.** Higher magnification of B1. The sheets of positive, moderate to strong immunolabeled round cells are multifocally interrupted by larger, rounded, strongly immunolabeled cells (possible local unidentified lymphoid cells, such as NK cells). **B3) Intestine.** Gut-associated lymphoid tissue (GALT) T-cells demonstrate membranous, cytoplasmic reactivity. **B4) Haired skin**. High magnification of B3. Matrical melanocytes display strong immunoreactivity to the target antigen. Follicular keratinocytes and adipocytes are negative to the target antigen (internal negative controls).

C) Male dunnart 31 (second batch). C1) Prostate. Random, moderate to strong membranous, perinuclear to cytoplasmic immunolabeling of leukocytes (possibly tissue macrophages) located in the inter-glandular stroma. Faint to light, random, extracellular and intracellular - non-specific staining is observed within the apical portion of columnar cells and glandular lumina (background staining). **C2) Prostate**. High magnification image of C1. **C3) Haired skin.** Similar to Dunnart 33, the skin contains a marked infiltration by round cells arranged in sheets; all negative to the CD3 leukocytic marker. A scattered population of larger, rounded cells contain cytoplasmic immunolabeling with CD3 (possibly tissue macrophages). **C4) Haired skin.** Higher magnification of C3. No immunolabeling is observed within keratinocytes (upper right corner) or dermal fibroblasts and collagen. Minimal cytoplasmic labeling is observed within mast cells (confirmed via toluidine blue).

Control tissue: Canine tonsil. Tonsillar tissue of both epithelial cell lineages and lymphatic lineages, serving as positive control for lymphatic markers and negative control for lymphatic markers. Tumours are avoided due to the variable expression of markers.

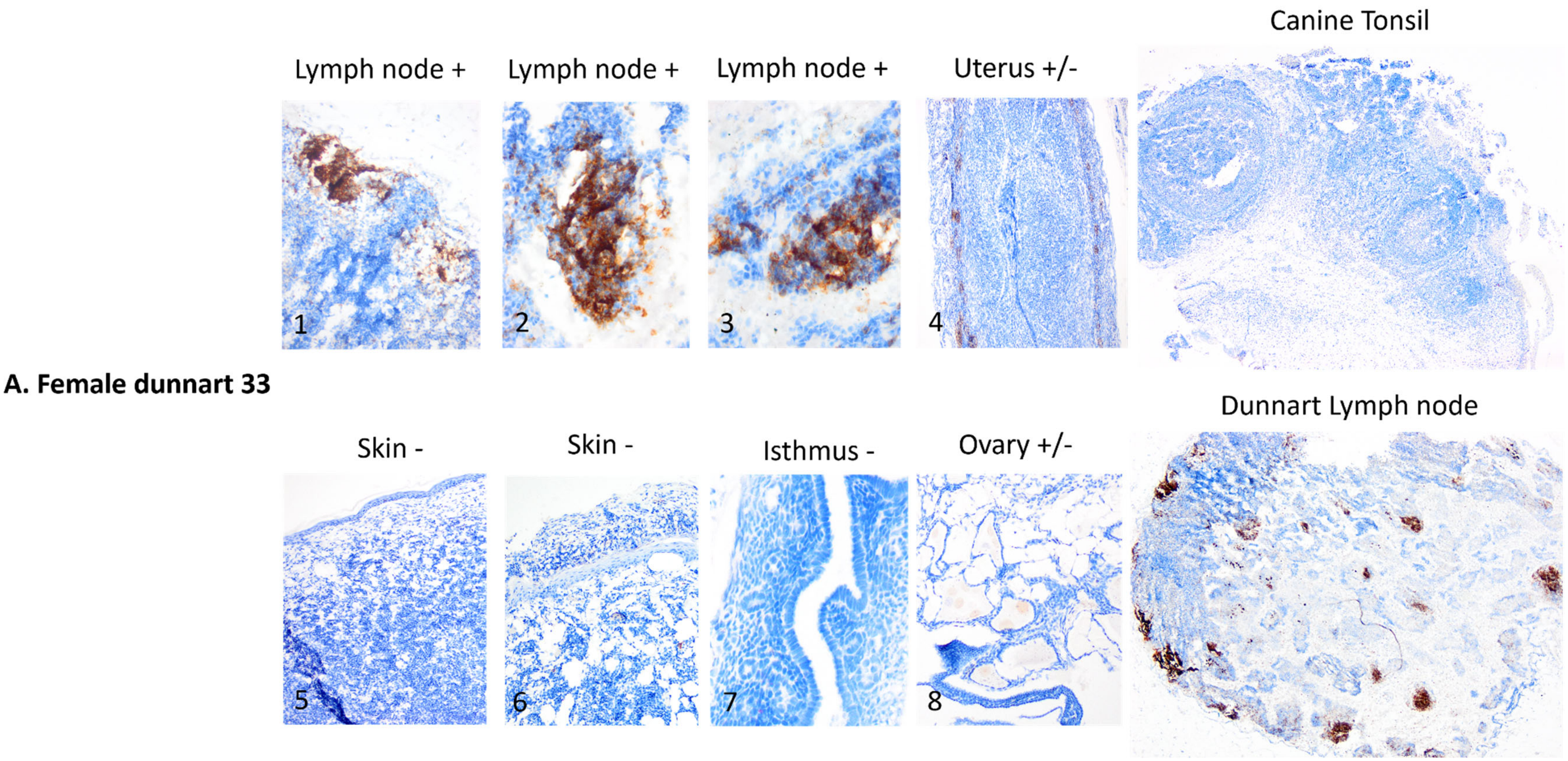

Supplementary Figure 2. Investigation into the immunolabeling of CD20 (membranous) leukocytic marker in Julia Creek dunnart tissue. All tissues belong to dunnart 33 (second batch).

A) Female dunnart 33 (second batch).

A1) Lymph node. Follicular structures show reactivity, evenly spaced out from a lymphoid population lacking reactivity, likely corresponding to paracortical T-cells.

A2) Lymph node. Higher magnification of A1. Focal membranous to cytoplasmic reactivity is observed within a lymphoid follicle, likely detecting pre-plasma cell differentiation, mature B cells and possibly follicular dendritic cells.

A3) Lymph node. Higher magnification of A2.

A4) Uterus. The myometrium shows a lack of reactivity, except for faint, random, non-specific reactivity of smooth muscle myocytes within *stratum vasculare*.

A5) Haired skin. The dermis is markedly expanded by an infiltrating population of round cells forming sheets that shows a diffuse lack of reactivity.

A6) Haired skin. Higher magnification view of an upper epidermis expanded with round cells arranged in sheets. The dermal collagen, fibroblasts, epidermis and overlying serocellular crust covering an eroded epidermis show a lack of reactivity. **A7) Oviduct (isthmus)**. There is diffuse lack of reactivity of the smooth muscle myocytes and epithelium.

A8) Ovary. There is diffuse lack of reactivity in the stroma and follicular epithelium. Faint, non-specific, extracellular reactivity is observed infrequently within ovarian follicles.

Control tissue: Canine tonsil and dunnart 33 lymph node. Canine tonsil tissue was added although CD20 has not been validated in this species, hence the lack of reactivity.

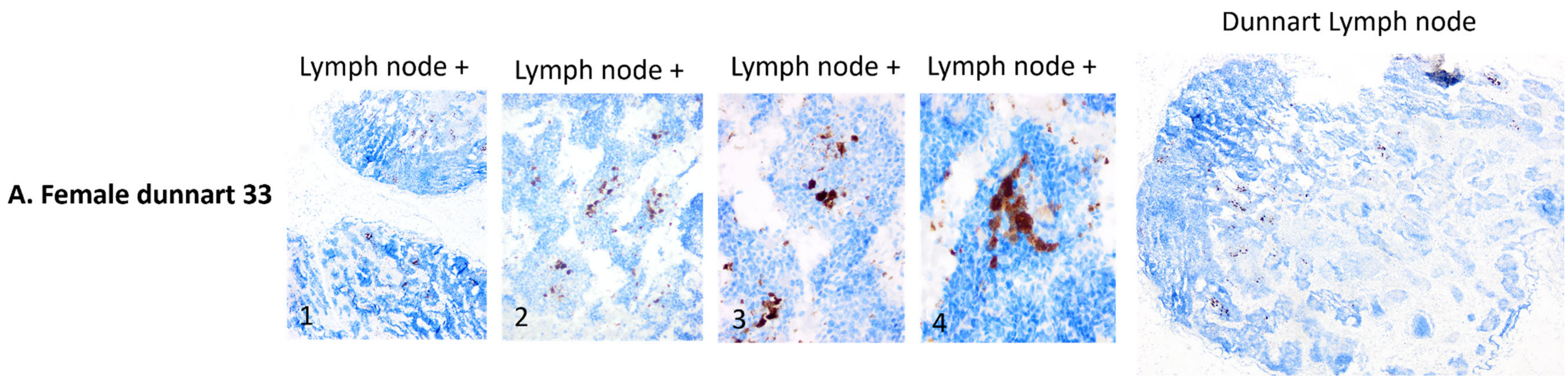

Supplementary Figure 3. Investigation into the immunolabeling of CD45 (membranous and cytoplasmic) leukocyte common antigen (LCA) marker in Julia Creek dunnart tissue.

A) Female dunnart 33 (second batch).

A1) Lymph nodes. Subgross view of cortex and medulla of two lymph node profiles with internodal stroma. Most of the cortex, all the paracortex, subcapsular and medullary sinuses and internodal stroma show a diffuse lack of reactivity. Within the cortex, a few scattered follicles have small numbers of reactive cells.

A2) Lymph nodes. Higher magnification image of cortical and paracortical tissue of the lymph node in A1 (upper right corner).

A3) Lymph nodes. Higher magnification image of cortical and paracortical tissue of lymph node in A1 (bottom). Reactivity is observed within the membrane and cytoplasm of rounded cells, which could correspond to nucleated cells of haemopoietic origin (lymphocytes, monocytes, macrophages).

A4) Lymph nodes. Higher magnification image of cortical and paracortical tissue of lymph node in A1 (bottom).

Control tissue: Dunnart 33 lymph node.

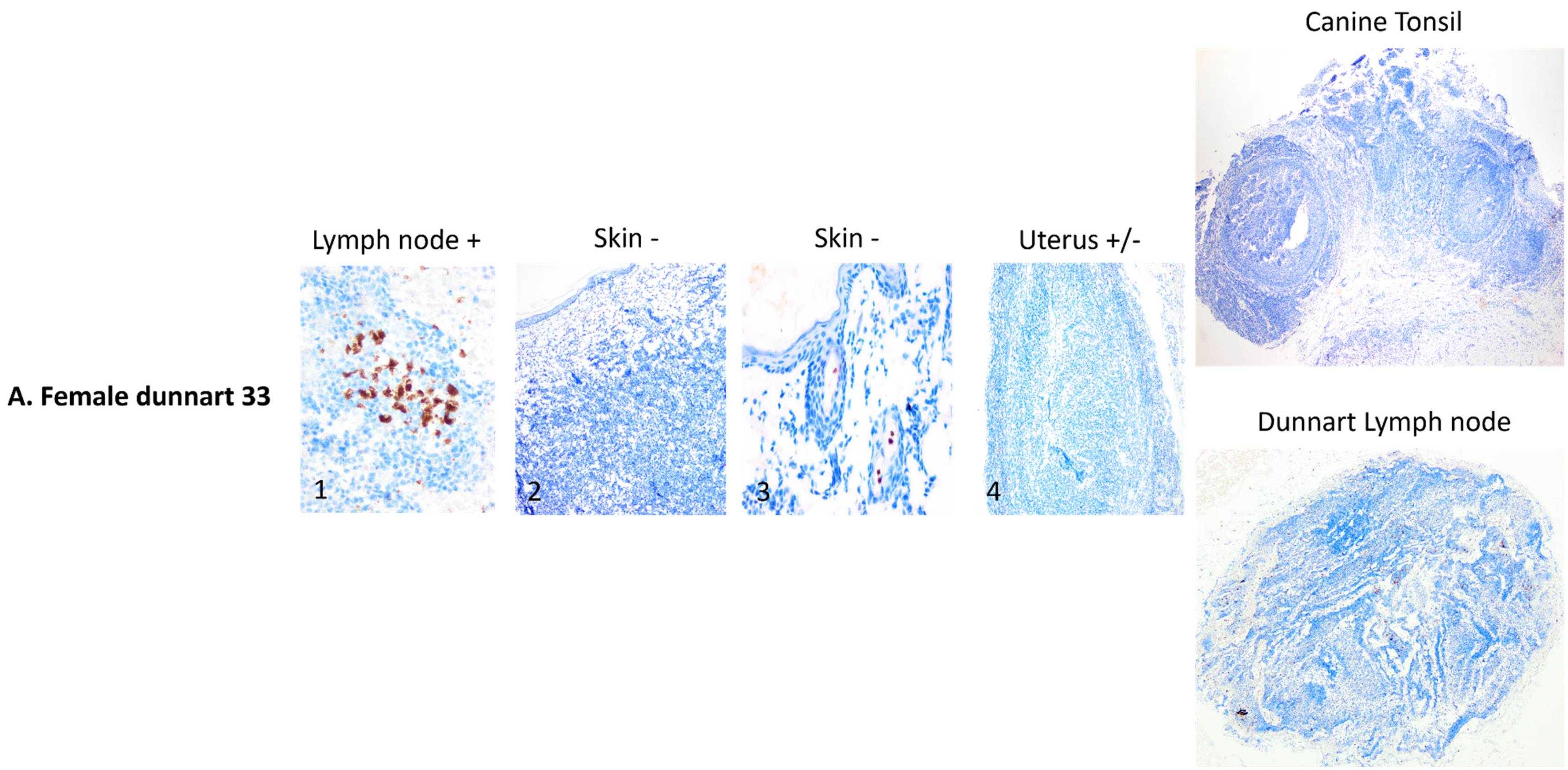

Supplementary Figure 4. Investigation into the immunolabeling of CD68 (cytoplasmic) leukocytic marker in Julia Creek dunnart tissue.

A) Female dunnart 33 (second batch).

A1) Lymph node. Small clusters of reactive rounded cells are observed within cortical nodal tissue. The typical reaction pattern of dot-like granular or diffuse cytoplasmic reactivity is observed.

A2) Haired skin. The round cell population expanding the dermis shows a diffuse lack of reactivity.

A3) Haired skin. Higher magnification view of the epidermis, dermal collagen, fibroblasts, and follicular epithelium with a diffuse lack of reactivity.

A4) Uterus. The myometrium displays a diffuse lack of reactivity (internal control).

Control tissue: Canine tonsil and dunnart 33 lymph node. Canine tonsil tissue was added although CD68 has not been validated in this species, hence the lack of reactivity.

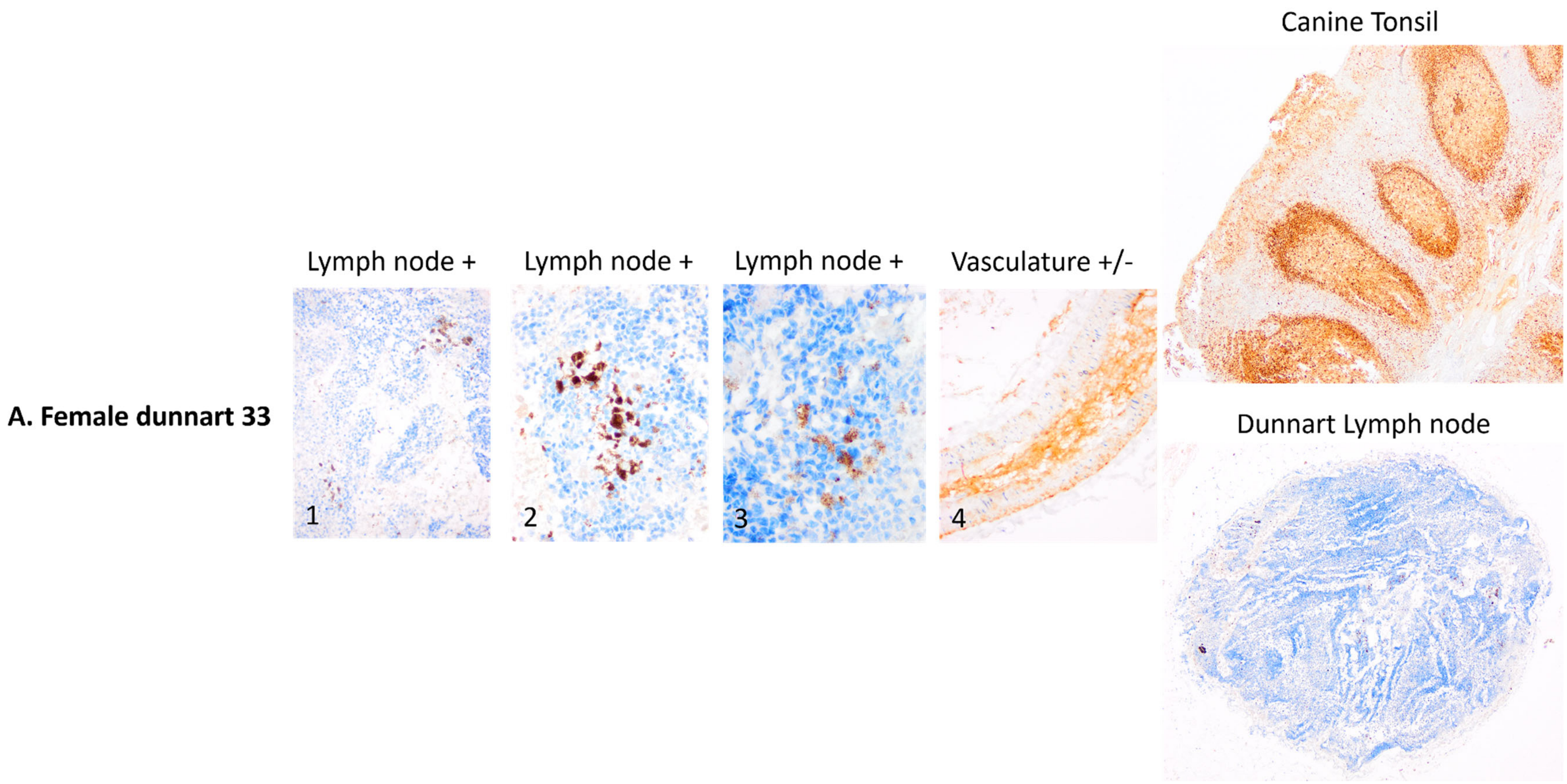

Supplementary Figure 5. Investigation into the immunolabeling of CD79a (cytoplasmic or membranous) leukocytic marker in Julia Creek dunnart tissue.

A) Female dunnart 33 (second batch).

A1) Lymph node. Two cortical lymphoid follicles display small clusters of reactive cells.

A2) Lymph node. Higher magnification image containing a small cluster of cells displaying cytoplasmic reactivity is observed within a nodal cortical follicle, possibly identifying some pre-pro B cell or plasma cells.

A3) Lymph node. Higher magnification image nothing reactivity is predominantly cytoplasmatic.

A4) Medium caliber vessel. There is non-specific, moderate reactivity within smooth muscle myocytic sarcoplasm and extracellularly within the vascular lumen.

Control tissue: Canine tonsil and dunnart 33 lymph node.

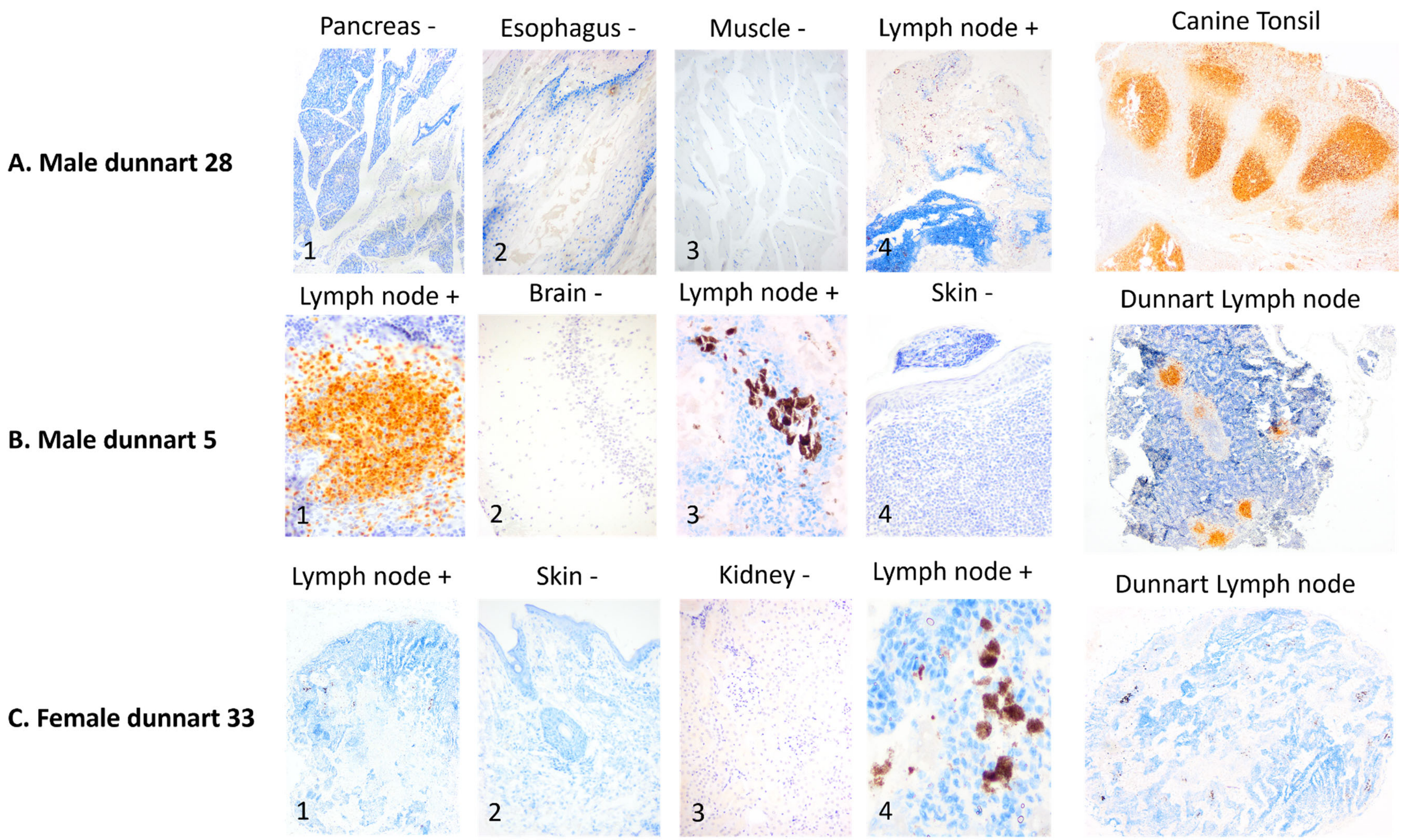

Supplementary Figure 6. Investigation into the immunolabeling of PAX5 (nuclear) leukocytic marker in Julia Creek dunnart tissue.

A) Male dunnart 28 (second batch). A1) Pancreas. There is diffuse lack of nuclear reactivity within exocrine and endocrine pancreatic tissue. Epithelium lining a pancreatic ducts also lacks reactivity (top right corner). **A2) Esophagus.** The parakeratinized epithelium lacks reactivity. Faint,

non-specific, extracellular immunolabeling is observed in the esophageal lumen. **A3) Skeletal muscle.** There is diffuse lack of nuclear reactivity within myocyte nuclei and satellite cells.

A4) Lymph node. Scattered, non-specific, reactivity is observed within perinodal tissue. **Control tissue:** Canine tonsil.

B) Male dunnart 5 (first batch). B1) Lymph node. High magnification image of a single lymphoid follicle displaying strong nuclear reactivity, likely identifying pre-pro-B cell through mature B cells. **B2) Brain.** High magnification image containing a section of grey matter with hippocampal neurons, all lacking reactivity. **B3) Kidney**. High magnification image containing a section of renal cortex with tubules and glomeruli lacking reactivity. **B4) Haired skin.** The dermis is expanded by round cells arranged in sheets. A small serocellular crust is observed overlying a focally eroded epidermis. No nuclear reactivity is observed in any cell population. **Control tissue:** Dunnart 5 lymph node.

C) Female dunnart 33 (second batch). C1) Lymph node. Subgross view of a second lymph node, in which small, scattered clusters of reactivity are observed. **C2) Haired skin.** The dermis is infiltrated by round cells that lack nuclear reactivity. Dermal fibroblasts, collagen, follicular and epidermal epithelium also lack nuclear reactivity (internal negative control). **C3) Kidney**. In the renal cortex, glomeruli and tubular epithelium lack nuclear reactivity (internal negative control).

C4) Lymph node. Higher magnification image of C2.

Control tissue: Dunnart 33 lymph node.

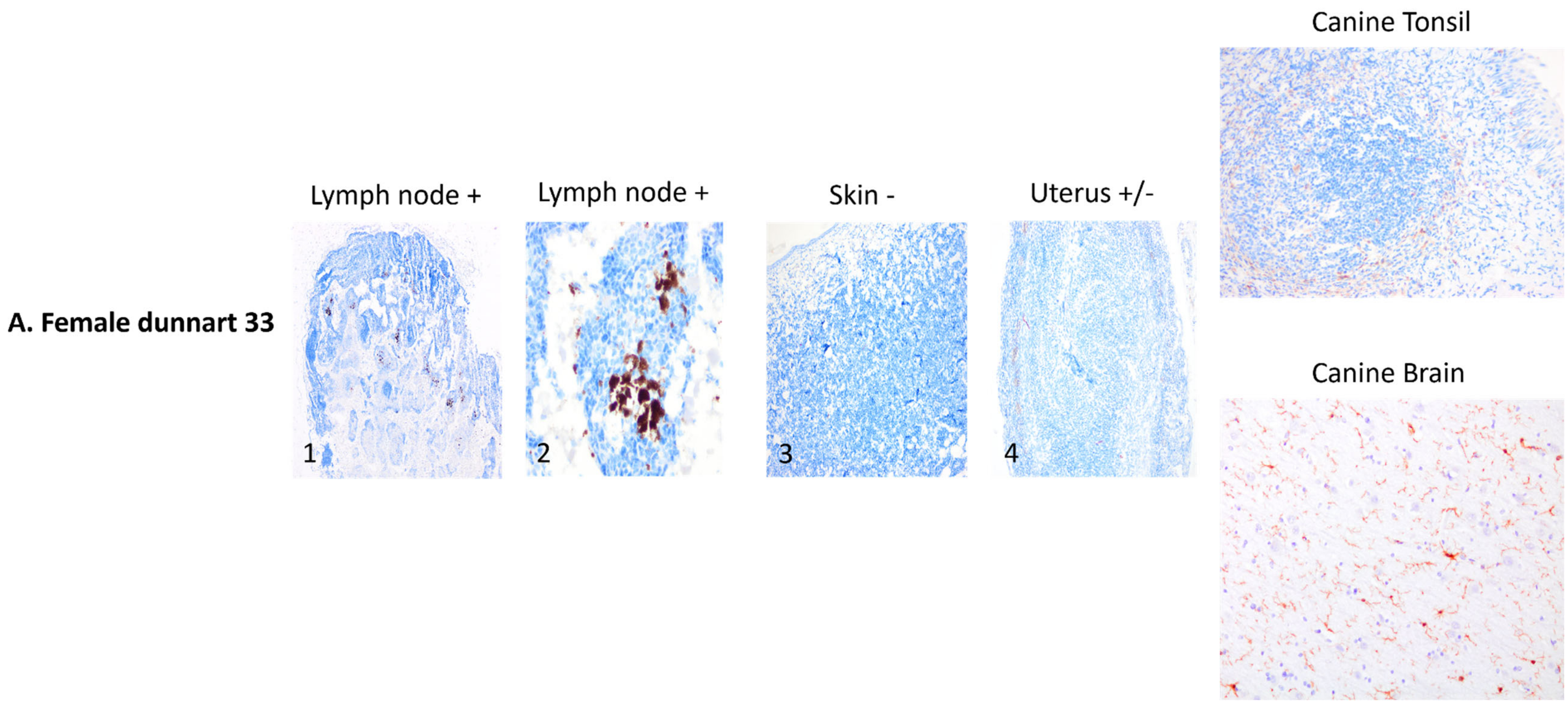

Supplementary Figure 7. Investigation into the immunolabeling of Iba-1 (cytoplasmic) histiocytic marker in Julia Creek dunnart tissue.

A) Female dunnart 33 (second batch).

A1) Lymph node. Low magnification image of control lymph node where clusters of cells displaying cytoplasmic reactivity are observed within multiple lymphoid follicles.

A2) Lymph node. High magnification image of control lymph node on A1.

A2) Haired skin. Diffuse lack of reactivity is observed in a round cell population infiltrating and markedly expanding the dermis.

A3) Uterus. Smooth muscle myocytes lack cytoplasmic reactivity. Faint, multifocal, non-specific, extracellular reactivity is observed within the stratum vasculare.

Control tissue: Canine tonsil and canine brain.

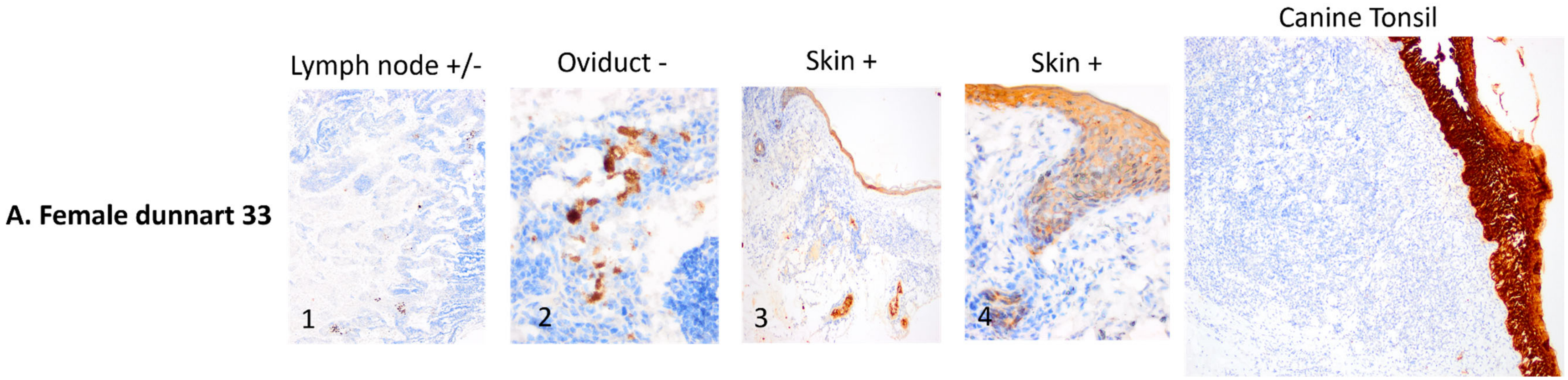

Supplementary Figure 8. Investigation into the immunolabeling of cytokeratin AE1/AE3 (cytoplasmic) marker in Julia Creek dunnart tissue.

A) Female dunnart 33 (second batch).

A1) Lymph node. Scattered cytoplasmic reactivity is observed in small groups of cells centering lymphoid follicles. Outer follicular cells and paracortical cells diffusely lack reactivity.

A2) Lymph node. High magnification view of A1.

A3) Haired skin. Moderate to strong cytoplasmic reactivity is observed within follicular and epidermal keratinocytes.

A4) Haired skin. High magnification view of the epidermis with moderate cytoplasmic reactivity within keratinocytes, and diffuse lack of cytoplasmic reactivity in the round cell population infiltrating and expanding the dermis.

Control tissue: Canine tonsil.
