# Supplementary figures and images for: Spontaneous Lesions of Endangered Geriatric Julia Creek Dunnarts (Sminthopsis douglasi, Archer 1979) with Emphasis in Reproductive Pathology
Source: Vet Sci. 2024 Mar 22;11(4):142. doi: 10.3390/vetsci11040142 (PMC11054575; doi:10.3390/vetsci11040142)

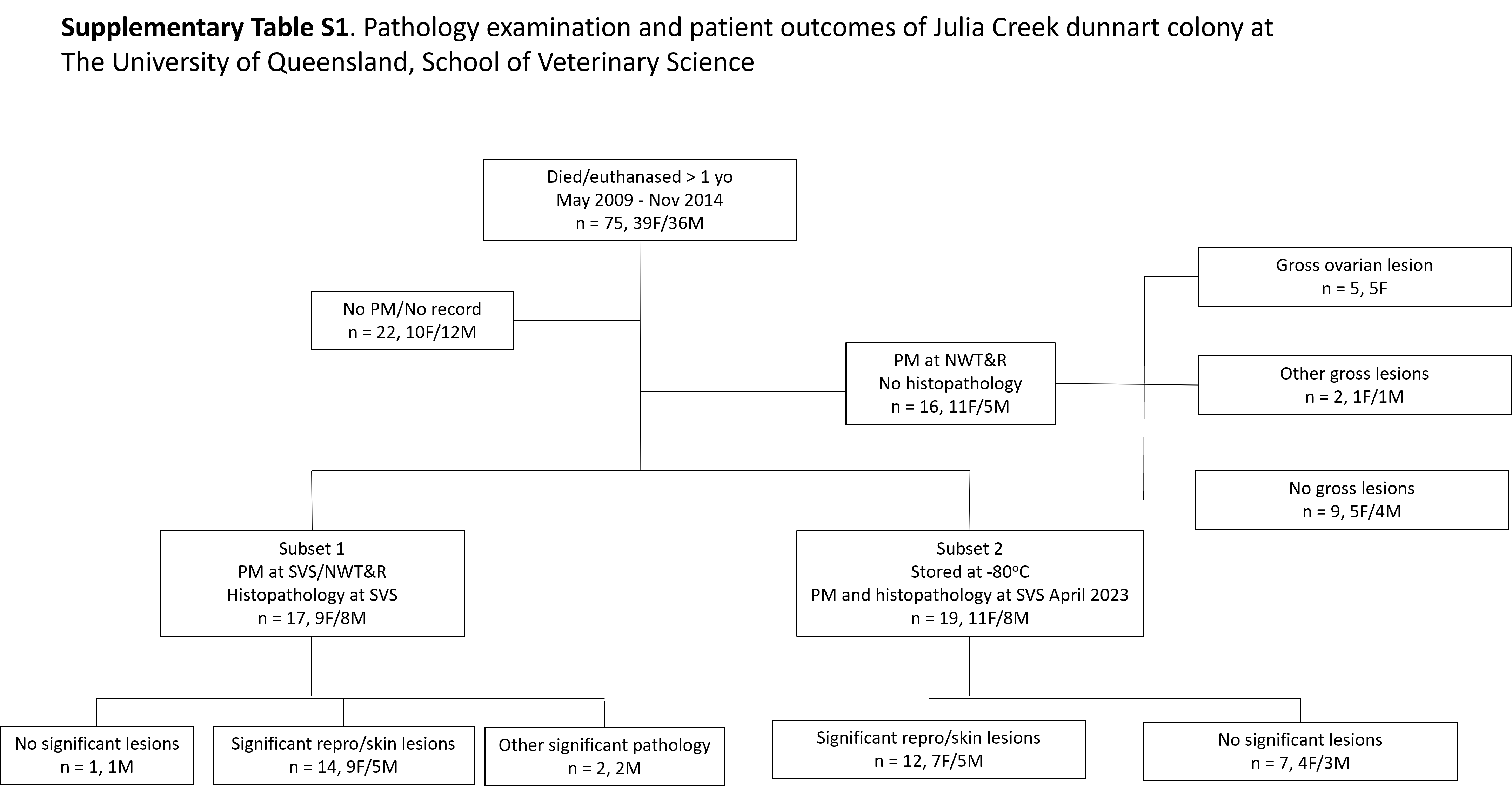

Supplement: Supplementary file 1 [file vetsci-11-00142-s001.zip › Supp Table S1.tif]
